# Unique small RNA signatures uncovered in the tammar wallaby genome

**DOI:** 10.1186/1471-2164-13-559

**Published:** 2012-10-17

**Authors:** James Lindsay, Dawn M Carone, Judy Brown, Laura Hall, Sohaib Qureshi, Sarah E Mitchell, Nicholas Jannetty, Greg Hannon, Marilyn Renfree, Andrew Pask, Michael O’Neill, Rachel O’Neill

**Affiliations:** 1 Department of Molecular and Cell Biology, University of Connecticut, Storrs, CT 06269, USA; 2 Department of Computer Science and Engineering, University of Connecticut, Storrs, CT, 06269, USA; 3 Department of Cell Biology, University of Massachusetts Medical School, Worcester, MA, 01655, USA; 4 Department of Allied Health Sciences, University of Connecticut, Storrs, CT, 06269, USA; 5 Cold Spring Harbor Laboratory, Cold Spring Harbor, NY, 11724, USA; 6 Australian Research Council Centre of Excellence in Kangaroo Genomics, Victoria, Australia; 7 Department of Zoology, The University of Melbourne, Victoria, 3010, Australia

## Abstract

**Background:**

Small RNAs have proven to be essential regulatory molecules encoded within eukaryotic genomes. These short RNAs participate in a diverse array of cellular processes including gene regulation, chromatin dynamics and genome defense. The tammar wallaby, a marsupial mammal, is a powerful comparative model for studying the evolution of regulatory networks. As part of the genome sequencing initiative for the tammar, we have explored the evolution of each of the major classes of mammalian small RNAs in an Australian marsupial for the first time, including the first genome-scale analysis of the newest class of small RNAs, centromere repeat associated short interacting RNAs (crasiRNAs).

**Results:**

Using next generation sequencing, we have characterized the major classes of small RNAs, micro (mi) RNAs, piwi interacting (pi) RNAs, and the centromere repeat associated short interacting (crasi) RNAs in the tammar. We examined each of these small RNA classes with respect to the newly assembled tammar wallaby genome for gene and repeat features, salient features that define their canonical sequences, and the constitution of both highly conserved and species-specific members. Using a combination of miRNA hairpin predictions and co-mapping with miRBase entries, we identified a highly conserved cluster of miRNA genes on the X chromosome in the tammar and a total of 94 other predicted miRNA producing genes. Mapping all miRNAs to the tammar genome and comparing target genes among tammar, mouse and human, we identified 163 conserved target genes. An additional nine genes were identified in tammar that do not have an orthologous miRNA target in human and likely represent novel miRNA-regulated genes in the tammar. A survey of the tammar gonadal piRNAs shows that these small RNAs are enriched in retroelements and carry members from both marsupial and tammar-specific repeat classes. Lastly, this study includes the first in-depth analyses of the newly discovered crasiRNAs. These small RNAs are derived largely from centromere-enriched retroelements, including a novel SINE.

**Conclusions:**

This study encompasses the first analyses of the major classes of small RNAs for the newly completed tammar genome, validates preliminary annotations using deep sequencing and computational approaches, and provides a foundation for future work on tammar-specific as well as conserved, but previously unknown small RNA progenitors and targets identified herein. The characterization of new miRNA target genes and a unique profile for crasiRNAs has allowed for insight into multiple RNA mediated processes in the tammar, including gene regulation, species incompatibilities, centromere and chromosome function.

## Background

Small RNAs play important roles in many aspects of pre- and post-transcriptional gene regulation, epigenetic modifications, chromosome segregation and genome structure. Small RNAs in mammalian cells have been categorized into different classes based on their size and biogenesis: 22 nucleotide (nt) microRNAs (miRNAs), 21-24nt endogenous short interfering RNAs (siRNAs), 26-32nt piwi interacting (piRNAs) (including repeat-associated siRNAs, rasiRNAs), and 35-42nt crasiRNAs (centromere repeat associated short interacting RNAs) (reviewed in [1-7]). Each class of small RNAs is synthesized by a distinct mechanism and each has discrete biological functions.

The first class of small RNAs identified were the micro RNAs (miRNAs), which are small (~22 nt) non-coding RNAs that regulate gene expression by base pairing to mRNAs where they direct either mRNA cleavage or repress translation [8]. Following a complex process of miRNA transcription, processing, and nuclear export, miRNAs are further processed by the RNaseIII enzyme, Dicer, and its cofactor TRBP. The mature miRNA is then loaded onto an Argonaute protein (Ago2 in humans) where it then interacts with and regulates the mRNA target. Confounding this, however, is the recent discovery that miRNAs can also function in gene activation through induction of promoter activity [9].

Another class of important small RNAs is the piRNAs. It has been proposed that piRNAs are synthesized by the sequential cleavage of long single stranded RNAs by members of the PIWI superfamily of proteins [2,10]. Importantly, piRNAs silence the expression of selfish repetitive elements in the germline [2,11,12] and appear to play a role in the establishment of heterochromatin through interactions with the PIWI family of proteins [3,13]. Moreover, piRNAs have recently been shown to play a key role in epigenetic gene regulation [14].

The crasiRNAs, originally discovered in the tammar wallaby, *Macropus eugenii*[15], are produced from transcription of repeats and are proposed to be essential components of cellular stability and chromosome segregation [16,17]. However, little is known about the biogenesis or sequence composition of these small RNAs. It is hypothesized that crasiRNAs emanate from both centromeric and euchromatic locations in the genome and may be involved in centromere specific histone recruitment [16,18].

The evolution of these different types of small RNAs can provide insight into both conserved regulatory networks as well as lineage-specific transcriptional regulation [19,20] that has been evolving independently from eutherian (mouse and human) mammals for over 160 million years [21]. This evolutionary distance makes the tammar an ideal model species for studying emergent specificities of small RNAs and their integration into regulatory networks that are mammalian, marsupial or tammar-specific. Furthermore, the tammar has several unique developmental innovations, including its hopping mode of locomotion, the development of a pouch, a short-lived and non-invasive placentation, the delivery of an altricial young, a lengthy and highly sophisticated lactation and *ex utero* sexual differentiation (reviewed in [22]), allowing for examination of small RNAs in the context of novel gene networks. Of note, the tammar is unique amongst mammals in that it provides a tractable model for the study of centromere structure at the genomic level due to the overall small size of the centromere and its lack of large, monomeric satellite arrays [15,16].

For this study, we used massively parallel sequencing to annotate and characterize the major small RNA classes in the tammar wallaby as part of the global effort to understand the genome biology of this Australian marsupial. Based on both the annotated Meug_1.0 assembly and the newly derived Meug_2.0 assembly [23], we developed a pipeline to identify miRNAs that are conserved in mammals as well as miRNAs that are novel to the tammar. In addition to a survey of testis piRNAs, we also present the first full annotation for crasiRNAs and compare their genome distribution to functional centromeric domains in the tammar genome.

## Results

### Library preprocessing

Pre-sequencing size restriction was performed on tammar pouch young brain, liver, testis, ovary and fibroblast cells to target the small RNAs in the 18-22nt range, encompassing the miRNAs. From testis total RNA, pre-sequencing size restriction targeted the small RNAs in the 28-32nt range, encompassing the piRNAs. In both pouch young testis and fibroblast cells, pre-sequencing size selection was performed to capture the small RNAs in the 35-42nt range, comprising the newly discovered crasiRNAs. Post sequencing processing was performed on 14,028,815 reads to clip, trim and verify accuracy of size selection for all three major size classes [23]).

The sequenced and filtered putative small RNAs from our datasets, along with the miRBase entries for every mature, annotated miRNA, were mapped against the tammar genome using an ungapped short read aligner (see methods). Each class of sequenced reads was further processed using our bioinformatics pipelines to filter noise and degraded products from bone fide small RNAs. Longer reference sequences such as repeats and hairpin precursors were mapped to the tammar genome using a gapped alignment tool similar to BLAST. Given the short length of the small RNAs and the expectation that at least some classes would be repeat-associated, we performed alignments reporting all valid mapping locations. Thus, all of our analysis strategies do not attempt to quantify the level of RNA in the experiment; rather, they simply measure presence and absence.

### Identification of miRNA genes

Our miRNA gene pipeline identified 21 putative miRNA genes, 13 of which have no known orthologs in other species and are therefore referred to as novel (Table 1). All of these contained intact open reading frames and were annotated as generic protein coding genes, and a further eight had detectable transcripts in whole embryo transcriptome datasets, indicating they are strong candidates for *de novo* miRNA genes in the tammar genome. The remaining eight of the 21 protein coding genes are annotated genes but were not considered to be miRNA genes according to Ensembl. Of these, six were detected as transcripts in embryo transcriptome datasets and a further four of these contained a high number of miRNA reads, classifying these as strong candidates for *de novo* miRNA genes in the tammar genome (Table 2). Included in these four are the genes *HOXD4* (described in [24]), *PANK3, NFYC*, and *CDC20B*. Finally 75 miRNA genes in the Ensembl annotation of the tammar wallaby genome were confirmed by our pipeline (Additional file 1: Table S1).

**Table 1:** **Previously unknown candidate miRNA genes identified in the tammar using Meug**_**1**.**0 annotations**

**miRNA count**	**Ensembl Meug 1**.**0 annotation**	**Symbol**	**Hairpin alignment**	**mRNA**
26	ENSMEUG00000003103	novel	..((((.((((..(((.(((((((((((((((.((.......)).))))))))…))))))).)))..))))))))…((((((((.....)))).))))	X
13	ENSMEUG00000016107	novel	..((((…(((((((.((((((((((.(((((((.((.......)).)))))))..))))))).....((((((...))))))))).))))))).))))…	X
13	ENSMEUG00000014431	novel	…(((.(((((....((((...))))))))).))).((((((((…))))))))…(((((((.(((.(((.......))).))).))..))))).....	
13	ENSMEUG00000013419	novel	...((((.(((...)))…))))....((.(((((((((((((((((((((.......)))))…)))).)).)))).)))))).))........	X
13	ENSMEUG00000010899	novel	.....(((((((((((((.(((..(((.....(((((.(((.(((((((((.......)))))))))))))))))))).))).)))))...))).)))))	
13	ENSMEUG00000010593	novel	.(((((.(((((..((((((.((((((((((((.((.......)).))))))…))))))...((.(((((…))))).))))))))...)))))…)))))	X
13	ENSMEUG00000010192	novel	.(((.(((…((((((((((…(((((.(((..........)))))))).))))))))))((.(((((…))))).))…((((...)))).))).)))..	
13	ENSMEUG00000008728	novel	.((((…(((((((.((((...((.(((......))).))…))))))))))).)))).((((((((((((((((.....)))))))..)))))))))...	
13	ENSMEUG00000005907	novel	(((.((.(((…((.(((((((((((.(((((((((.......))))).))))...)))))))…)))).)).))).)))))............	
13	ENSMEUG00000003443	novel	...((.(((((..))).)).))…((.((((((((.(((((((((…((((((((.(((.......))).)))))))))))))))...)).))).))))).)).	X
13	ENSMEUG00000002502	novel	.(((((…(((.((.((((((((((((.(((((((.((.......)).)))))))..))))))).((((..))))))))).)))))...)))))......	X
13	ENSMEUG00000001788	novel	((((..))))((((((.((((((((((((((((((.((.......)).)))))))))…)))))).((((.......))))....))).))))))...	X
13	ENSMEUG00000000143	novel	…(((((.(((.((((......)))).))).)))))(((((((…)))))))....(((((...((((.(((((((...))))))).))))...))))).	X

Note that there are no miRBase orthologs.

**Table 2:** Previously annotated protein coding genes predicted herein to be miRNA genes in tammar

**miRNA count**	**Ensembl Meug 1**.**0 annotation**	**Symbol**	**Hairpin alignment**	**mRNA**	**mirBase**
1389	ENSMEUG00000014939	PANK3	((((.(((((((((((.((.(((((((((((.(((......)))))))))))))).)))))).))))))).))))........(((((((......))))))).	X	age-mir-103
1145	ENSMEUG00000004480	NFYC	((((((.((..((((...)))).))))))))(((((((.((((((((((((((((.(((((((((((........)))))))))))))))))))))))))))))))))).	X	eca-mir-30e
554	ENSMEUG00000000911	CDC20B	......((((.(((…))).))))..((.(((((((((((((((((((((.((.(((((((........))))))))).)))).))))))))))))))))).)).	X	
349	ENSMEUG00000016575	HOXD4	.(((.(((((((....))).)))).))).(((((.((((((((.((((.(((((.((((((((((........)))))))))).))))))))).)))))))).))))).	X	aca-mir-10b
79	ENSMEUG00000012110	PFDN5	…((((((((((((...((((.((.......)).))))))))))((((((((.((...)).))))))))...)))))).....((((...)))).		
26	ENSMEUG00000012937	BCAS3	......((((.((((((((.......)))))))).))))(((((((.(((…((.((.(((…(((((((…)))))))..))).)).)).)))))))))).		
26	ENSMEUG00000008344	MYOZ2	(((((((.((((.((.(((((((((((((.......((.((((…(((((((......))))))))))).))))))))))))))).)))))).)))…)))).	X	
26	ENSMEUG00000004683	GRIA1	...(((...(((((.((((((((((.(((((((.((.......)).)))))))..))))))).(((.((((…)))))))...))))))))....)))..	X	

For each, the number of miRNA reads, identification of mRNA transcripts in embryos and any identified miRBase orthologs are indicated.

**Figure 1:**
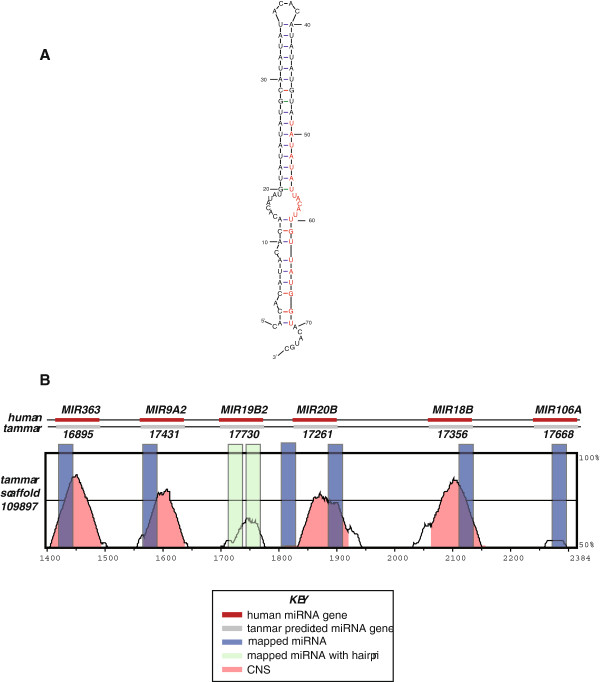
**X-linked miRNA genes in tammar. A**. Hairpin prediction for miRNA ENSMEUG00000017730. **B**. Vista plot of alignment between human X miRNA gene cluster and tammar annotated miRNA gene cluster. Percent identity between human and tammar at any given region. High identity among sequences (70% across the sliding window) are indicated in red as conserved non-coding sequence (CNS), tammar miRNA alignments are shown in blue and hairpin-derived miRNAs are shown in green. INSET is key to annotations.

One significant overlap between the Ensembl annotation and our pipeline lies within a region orthologous to a miRNA gene cluster on human Xq26.2 (133,303,269-133,304,396bp). The human cluster contains six miRNAs (MIR363, MIR19A2, MIR19B2, MIR20B, MIR18B and MIR106A), all six of which were predicted from Meug_1.0 (ENSMEUG000000: 16895, 17431, 17730, 17261, 17356, and 17668 respectively). All carry high sequence identity between human and tammar with the exception of MIR19B2 and MIR106A, which carry low sequence identity (i.e. less than the threshold of 70% across the sliding window). Surprisingly, the only miRNA gene within the cluster for which a hairpin was predicted is the tammar ortholog to MIR19B2 (Figure 1A), a ncRNA gene with low sequence identity; however, all miRNA genes in this cluster carried an aligning miRNA within our datasets (Figure 1B). Additionally, another miRNA was found between MIR19B2 and MIR20B that is in a region of low homology between human and tammar yet outside of any predicted gene. This region may represent a unique site within tammar where a miRNA targets specific miRNA genes for regulation (Figure 1B). Interestingly, there is enrichment for this mature miRNA in our testis pool, indicating there may be testis-specific de-regulation of genes the MIR20B produced miRNA typically silences.

### Conservation of miRNA targets

To identify miRNA target genes that may be under post-transcriptional regulation in the tammar, valid miRNA alignments not contained within a hairpin structure were surveyed. A total of 163 genes were identified using Meug_1.0 annotations as potential targets for miRNA regulation. The vast majority of these were found within testes (145), with the remainder shared among ovary, brain, liver and fibroblast datasets (51, 12, 47, and 64 respectively). Each target gene identified in our dataset, along with the number of unique miRNA reads to that target, was cross-referenced with the miRanda database of target genes to identify both conserved and novel miRNA regulated genes in the tammar [25]. The microRNA.org database contains the alignment and score of sequences from miRBase mapped to various genomes (e.g. human, mouse, drosophila). The miRNA tissue-specific pools sequenced for the tammar were used as an analog to the miRBase sequences and counts of alignments to genes were generated using the short read alignment tool Bowtie (see methods). The intensity of each gene is indicative of how many sequences from the database (miRBase for human, mouse, drosophila and the individual mapped miRNAs for tammar) are attributed to that gene, but is not a proxy for the quantitative measure of the abundance of miRNAs. This view of miRNA targets across multiple species was used to identify conserved and novel miRNA genes, and to place a loose confidence on the accuracy of the putative microRNA targets in tammar.

**Figure 2:**
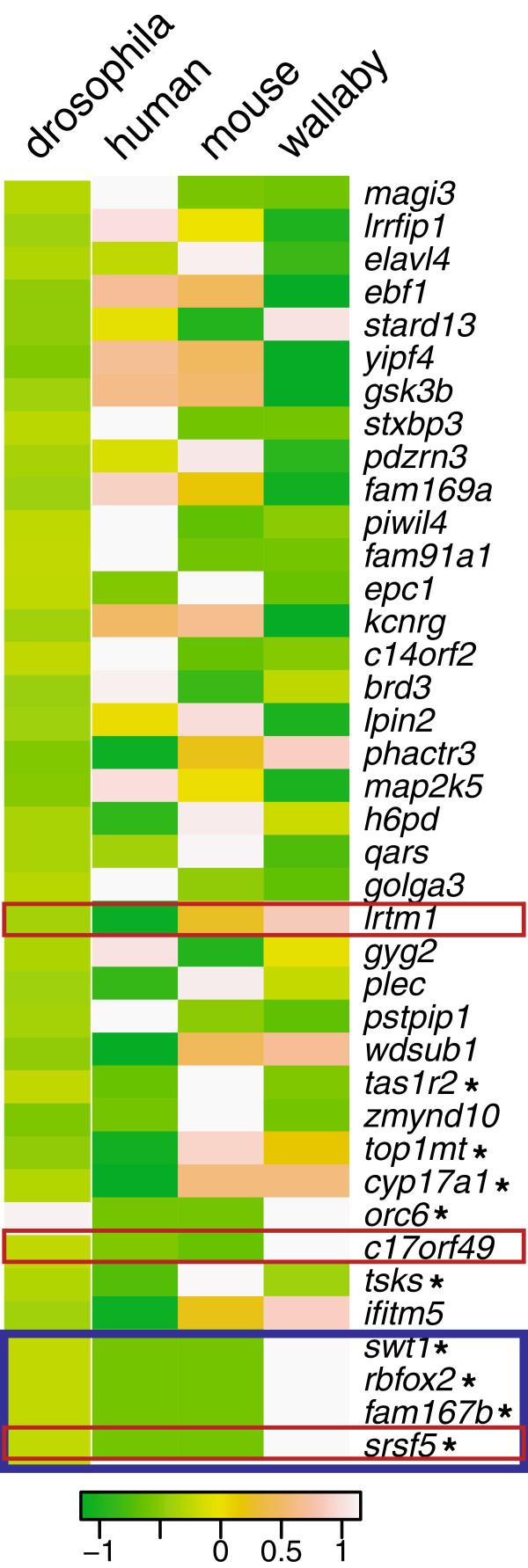
**A heat map indicating abundance of miRNA targets between miRBase for drosophila, human, mouse and sequenced pools for tammar. **The map is normalized by row with darkest green indicating no hit, and white indicating high density of hits to miRBase. Genes outlined in red are those shown in detail in Figure 3. The genes outlined in blue are those that have a miRNA only in tammar, the genes indicated with an asterisk have no orthologous miRNA in human.

From these analyses, nine genes were identified in tammar that are novel miRNA regulated genes when compared to human, although four share conserved miRNAs with mouse and one shared a conserved miRNA only with drosophila. The final four of this set of genes do not carry resemblance to any previously annotated miRNA targets (Figure 2). Tammar genes with high intensities relative to other tammar genes on the heat map presented in Figure 2 provide some indication of confidence that these genes are indeed miRNA targets; unfortunately, other factors such as low coverage and tissue specific expression may account for tammar genes with lower intensities. Specific genes were targeted for further comparison based on variations in density of miRNA reads between tammar, mouse and human in an effort to illustrate the utility of tammar as a means to identifying novel miRNAs within other species as well as tammar-specific miRNAs.

**Figure 3:**
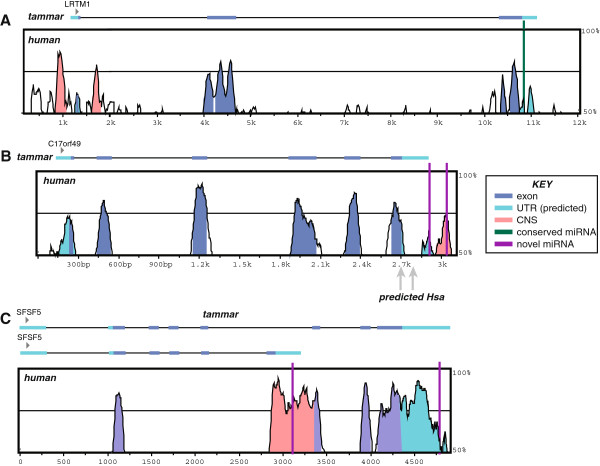
**Vista plot of alignment between human and tammar miRNA target genes.** Percent identity between human and tammar at any given region. High identity among sequences (70% across the sliding window) is indicated in red as conserved non-coding sequence (CNS), blue as exons and light blue as 3^′^UTR (with the exception of the intronic UTR for the alternatively spliced form, which is indicated in red). Conserved and novel miRNAs as indicated in key INSET. **A**. Annotation for *Lrtm1*. **B**. Annotation for C17Orf49. Predicted miRNAs in human (Hsa) are indicated. **C**. Annotation for *Srsf5*.

As an example, *Lrtm1*, leucine-rich repeat and transmembrane domain-containing protein 1, is a gene with a high density of miRNA reads in tammar and mouse, but a very low density in human (69, 49 and 3, respectively). Vista alignment between human and tammar indicate this gene has a highly conserved exon structure between these two species, with a conserved miRNA target in the 3‘UTR (Figure 3A).

In contrast, the gene C17orf49, like *Lrtm1*, has a conserved intron-exon structure between tammar and human (Figure 3B), yet the predicted miRNA target sites are not conserved. In human and mouse, there are virtually no miRNA target sites in this unknown gene (8 miRNAs that map to two predicted sites in human and 0 miRNAs in mouse), yet there are 136 miRNAs that map to two unique target sites in the 3’UTR. The majority of these miRNAs target a second site in the 3’UTR that is also highly conserved between human and tammar (CNS in Figure 3B). In yet another example, *Srsf5*, we have identified brain-specific miRNAs for a single target site that are tammar-specific. This gene contains no predicted or verified miRNAs from any other species (including human, mouse, rat, fruitfly and nematode) (Figure 3C). *Srsf5* is annotated in the human genome as two alternatively-spliced transcripts, with only a few of the exons from either transcript annotated in Meug_1.0 due to low sequence coverage of this region. However, the 3’ exons and 3’UTRs for both alternative transcripts are well annotated and share high identity between mouse and human. Both tammar miRNA targets fall within the 3’UTRs, one in each of the two alternatively spliced transcripts. The shorter transcript variant contains a miRNA that falls within a very conserved region of the 3’UTR while the second miRNA falls within a region of much lower identity within the 3’UTR of the longer transcript variant (Figure 3C).

### Mobile DNA and piRNAs of the tammar

**Figure 4:**
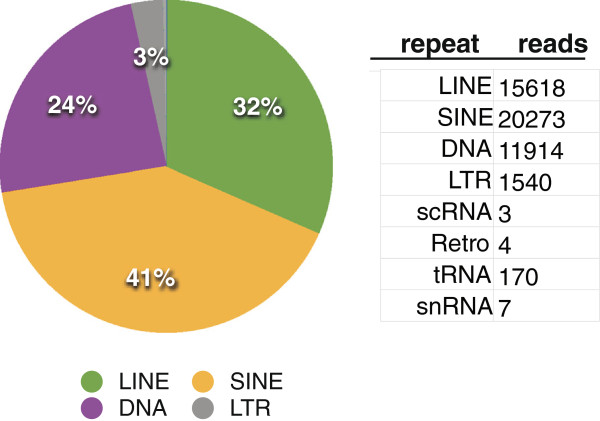
**Annotations for the piRNAs from tammar testis. **To the left is the relative distribution of annotations shown in the table to the right. The table indicates the number of reads that overlap with a specific element class.

We identified piRNAs from pouch young testis. After clipping and trimming, piRNAs from the testis pool were mapped to the tammar genome assembly Meug_2.0. Note that while assembly 1.1 contained gene annotations, 2.0 contains comprehensive repeat annotations. The mapped locations of piRNAs were then compared for overlap with known repeats as annotated by Repeat Masker [26] and novel repeats annotated by our in house repeat annotation pipeline [23]. piRNAs from the tammar, similar to those found in other species, are mobile element enriched. The vast majority of piRNAs are derived from LINEs and SINEs in the tammar (73%), followed by DNA elements (24%) and LTR-containing retroviruses, including KERV (3%) (Figure 4, Additional file 2: Table S2). Within the LTRs, ~4% map to LTR-elements unique to the tammar genome. While the genome assembly is too fragmented to assay for clusters of piRNA producing repeats, we confirmed that piRNAs in the testis are derived from both conserved repeats and tammar-specific repeated elements (specifically LTRs) (Figure 4).

### crasiRNA and the centromere of the tammar

**Figure 5:**
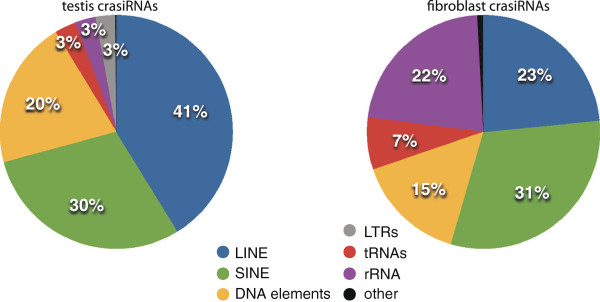
**CrasiRNAs are derived from repeats in tammar. **Relative distributions of repeat annotations, including both *de novo *and RepBase annotated repeats, for the crasiRNAs from tammar testis (left) and fibroblast cells (right). Key of elements at the bottom; “Other” includes all elements represented at <0.5%.

While the three major classes of small RNAs (siRNAs, miRNAs and piRNAs) and variants within each class (e.g. endo-siRNAs), have been well studied in various model systems, a fourth major class, crasiRNAs, was first identified in the tammar [15]. Named after the original elements characterized within the pool, this class of small RNAs is larger than those previously characterized, falling within a size range of 35-42nt, and appear to be derived from centromeric elements (centromere repeat associated short interacting RNAs)[15]. To determine whether this novel size class of small RNAs is indeed centromere-associated, we aligned all the crasiRNA sequences in the pool to annotated, *de novo*, and known centromeric repeats as well as to other repeated elements annotated in the tammar genome Meug_2.0 (Figure 5, Additional file 3: Table S3). This analysis indicates the crasiRNAs are enriched for repeated elements (LINEs, SINEs, transposons), although it was not possible to determine from this mapping scheme whether the repeat elements themselves were associated with centromere domains. However, the testis and fibroblast cell crasiRNA distribution is not identical, with a preponderance of LINE-derived crasiRNAs in the testis and SINE-derived crasiRNAs in fibroblast cells. To confirm that there was no overlap between the testis piRNA and testis crasiRNA pools, regardless of the size limitations performed in the small RNA sequencing and subsequent data analyses, we identified only 10 crasiRNAs that overlapped with seven piRNAs using the one mismatch mapping strategy (methods). Thus, these two classes are largely derived from similar classes of repeats, although the repeat loci themselves are different.

**Figure 6:**
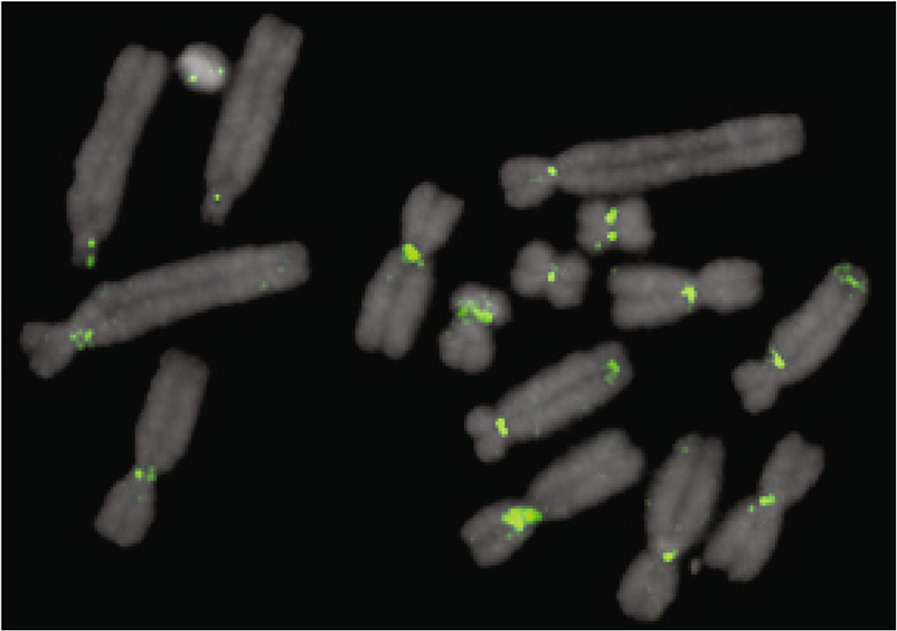
**Primed in situ hybridization using primers for crasiRNA pool sequence, SINE28 (green), to tammar metaphase chromosomes (grey). **SINE28 sequences are found localized to the tammar centromeres.

To verify centromere residence, crasiRNA sequences representative of elements that are highly abundant in the pool (SINEs, LINEs) and of lower abundance (LTRs, RTEs), as well as representative of different types of repeats (LINEs, LTRs, SINEs), were mapped to the tammar karyotype using primed in situ hybridization (PRINS). Over 80% of mapped crasiRNAs were found predominantly within centromere regions, with interstitial signals found at the telomeres and regions of the genome previously annotated as evolutionary breakpoints [27] (Figure 6, Additional file 4: Figure S1). Interestingly the crasiRNA with a high density of reads, derived from the newly annotated mammalian-specific SINE (SINE28), showed a strong centromeric signal (Figure 6), further supporting the hypothesis that crasiRNAs are derived from mobile elements found at active centromeres in the tammar karyotype.

**Figure 7:**
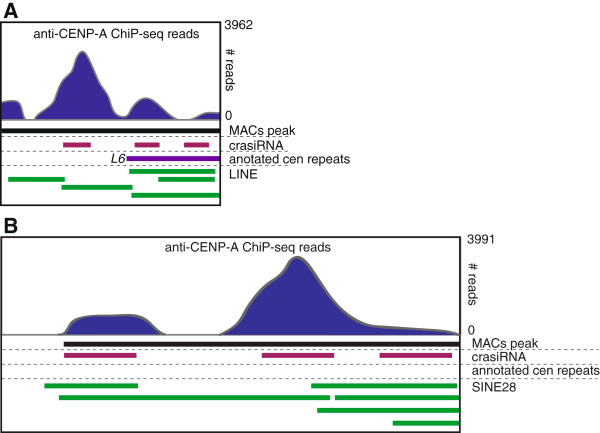
**Centromeric contigs in the tammar genome annotated with anti CENP A ChIP-seq coverage profile of number of reads (blue), MACs peaks (black),crasiRNA reads (red), previously annotated centromere (cen) repeats (purple) and annotated repeats (green). A**. Region of all elements co-mapping to a LINE. **B**. Region of all elements co-mapping to SINE28.

While our previous study showed that the original pool of small RNAs in the size range of 35-42nt, without separation based on annotation, did in fact co-localize to centromeres in the tammar [15], this new data confirms specificity of the individual sequence types within the crasiRNA pool. ChIP-seq with an antibody against tammar CENP-A, the modified histone specific to centromeres [28], provided further verification of centromere association. The ChIP-seq data set was co-mapped with repeat modeller annotations, crasiRNA pool sequences, contigs containing a high density of previously annotated centromere repeats, and previously annotated centromere repeats [27]. ChIP-seq peaks coincided with SINE, LINE and novel repeats within these contigs (Table 3, Figure 7A, B). Moreover, the densest peaks for the DNA bound to CENP-A nucleosomes were found in regions with the highest density of crasiRNA reads (Additional file 5: Figure S2). Across all centromere-annotated contigs, 93 of the 125 crasiRNA peaks identified overlapped with regions of CENP-A enrichment.

**Table 3:** **Distribution of ChIP**-**seq peaks with respect to the repeats found in centromeric contigs in the tammar assembly**

**Repeat class**	**CENP**-**A ChIP**-**seq peaks**
Simple	1
LTR/ERVK	2
LINE/RTE-BovB	6
DNA/En-Spm	9
DNA/Chapaev	14
SINE	16
Unknown satellite	18
LINE/CR1	22
LINE/L2	32
LINE/L1	193
SINE/MIR	195
buffer*	368

*de novo repeat.

### Sequence motif discovery for tammar crasiRNAs

**Figure 8:**
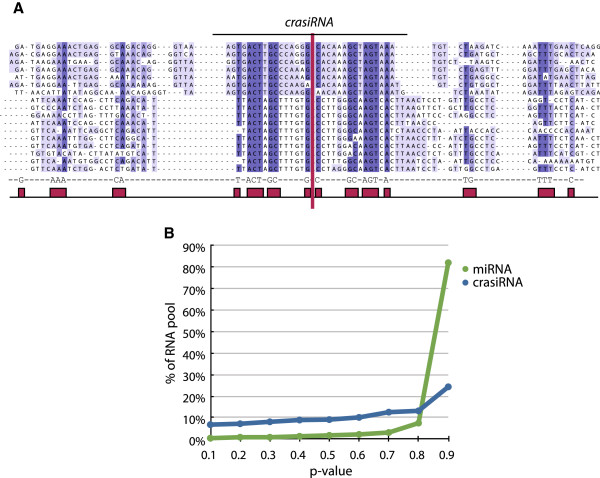
**A. Snapshot of an alignment of 284 crasiRNAs and surrounding sequence from the tammar genome. **The blue indicates conservation (white = 0%<dark blue= 100% identity) across all alignments. Shown at the bottom are the conserved nucleotides and boxes indicate the pattern of conservation. Listed from top are crasi-tam-001 through −017. The vertical red line bisects the crasiRNA. **B**. Distribution of statistical significance for the palindrome motif identified in crasiRNAs and miRNA pools.

In an effort to identify a sequence motif that might be shared amongst the crasiRNAs, regardless of their point of origin in the genome, we performed alignments [29] of 50bp up and downstream of all crasiRNA alignment locations in the tammar genome. For each crasiRNA which mapped to the genome multiple times, it was observed that the entire alignment window displayed high identity across all instances, regardless of the progenitor sequence. Conservation (100% identity) of specific nucleotides was uncovered across alignments with a distinct pattern within the crasiRNA and flanking sequences. This pattern is distinguished when each window is reported according to the strand the crasiRNA mapped to (sense or antisense) (Figure 8A). The motif is best described as a mirror pattern, or discontinuous palindrome, such that when the crasiRNA is split down the middle (see vertical red line in Figure 8A), each side of the crasiRNA and flanking sequence carries specific nucleotides that are complementary to one another (Figure 8A). This “mirror” pattern is shared among 63% of all crasiRNA loci (with at least ⅓ of the bases containing a complementary match).

A simple statistical significance test was developed to assign a p-value to each alignment and its flanking region. The score of a window represents the number of complementary matches between the sequence and its reverse complement. A p-value for this observation is computed by randomizing the sequence 100 times and observing the number of random tests that have a score equal to or greater than the original. A distribution of the p-values across the crasiRNA and miRNA pool (Figure 8B) indicates that this motif appears more frequently at higher confidences in the crasiRNA pool than expected at random. Moreover, this test shows that this motif is not specific to small RNAs in general, as it is not found in the miRNA pool. However, distributions for both miRNAs and crasiRNAs have a heavy tail, indicating many low confidence scores, which can be attributed to noise in the pools or sequence composition. For example, if we consider an AT-rich sequence, the probability of finding palindromic matches by chance is higher than a sequence with equal base composition across all four nucleotides. In the future, these concerns can be addressed by developing a more robust scoring and significance test that can capture higher order dependencies in the sequence. Since the crasiRNAs are derived largely from repeated elements, it would be interesting to explore enrichment of discontiguous palindromic motifs in specific regions of the genome such as those enriched in repetitive elements and centromeric regions.

## Discussion

### miRNA gene predictions

The presented pipeline identified 21 high quality, previously unknown miRNA genes in tammar using a strict gene annotation and confirmed 75 of the 421 known miRNA genes in tammar. The remaining miRNA genes predicted in Ensembl that do not match a mature miRNA from one of our datasets could be bone fide miRNA genes for which a mature miRNA is not expressed or sequenced in one of the target tissues analyzed herein. Alternatively, these could also represent miRNA loci that, while carrying sequence orthology to miRNAs in miRBase, have undergone lineage-specific locus death by genetic drift due to a lack of selection for function in this lineage [19]. However in light of our validation experiments and since each of the steps in our pipeline utilizes published tools, we have high confidence in our predictions.

Within our miRNA gene dataset are three pseudogenes that represent novel miRNA genes in the tammar. Previous work has shown that two miRNAs in primates were derived from processed pseudogenes [30], although the incidence of this type of miRNA gene evolution is considered rare [19,30]. Thus, there has been lineage-specific selection on the hairpins found in these pseudogene transcripts, which we can infer is involved in tammar-specific gene regulation given the mature miRNAs observed from these loci.

Closer examination of a cluster of miRNAs genes on the human X chromosome indicates there is high conservation of this specific miRNA gene cluster in metatherian mammals. This cluster is likely conserved on the X chromosome in tammar as it found on human Xq26.2, in a region on the ancient portion of the mammalian X chromosome and conserved on the X in marsupials [31,32]. While the conservation of the six miRNA genes in this region was confirmed by the presence of mature miRNAs in our miRNA pools, a miRNA peak was identified just downstream of MIR20B that was highly represented in the testis. The placement of this miRNA just adjacent to the 3’ end of this miRNA gene indicates this gene is likely under post-transcriptional regulation by a miRNA derived from another location, specifically in the testis. This would lead to a loss of gene regulation for targets of MIR20B in a testis-specific fashion, although the specific cell type affected and functional consequences remain to be determined.

### Mature miRNA analyses

For each of the microRNA pools, many of the miRNA reads did not overlap with known mature miRNAs annotated in miRBase, indicating that the tissues analyzed in the tammar may carry numerous novel microRNAs or that there has been high sequence divergence from previously annotated animal miRNAs. However, this may be an overestimation of lineage-specificity based on the criteria used in the mapping pipeline. Each RNA from miRBase, along with the sequenced miRNA pools, was mapped to the genome allowing for at most one mismatch to the genome sequence. This procedure indirectly performs an un-gapped alignment with no more than two mismatches between each miRBase annotation and sequenced tammar miRNA. While allowing more mismatches would increase the likelihood of identifying false miRNA targets, relying on such high stringency to identify conserved miRNAs may not account for deep evolutionary distances. This data will ultimately be used to develop new annotation methods that not only use direct information such as sequence similarity to previously annotated miRNAs, but also indirect information such as a predicted set of target genes.

Our annotation strategy for mature miRNAs allowed for assessment of target genes. While limited in the number of target genes to those with a full annotation in Meug_1.0, we were able to identify several tammar-specific miRNA targets, confirm conserved miRNA targets and potentially identify previously unknown miRNA targets in other species, such as human. For example, a conserved miRNA target was identified in the 3’UTR of the gene *Lrtm1* (Figure 3A), although the usage of this particular miRNA target varies across species (Figure 2). Thus, while miRNA utility may be species- or tissue-specific, the target location remains conserved. Within the annotated 3’UTR of C17ORF49, we identified two miRNA targets that appeared at first glance to be tammar-specific. However, closer examination of the conservation of this gene between tammar and human indicates these two locations are specific sites of high conservation, spanning ~160 million years of evolution. Note that the predicted human miRNA target sites are not correspondingly conserved (Figure 3B). The two tammar-identified target locations may indicate a conserved miRNA site in human that was previously unknown (Figure 3B). Moreover, C17ORF49 is a gene of unknown function in both tammar and human, thus indicating that the regulatory network of miRNA target genes may aide in understanding novel gene function.

Our analyses also identified several target genes that may represent tammar-specific miRNA regulation. One example of this was the gene *Srfs5* (Figure 3C), which carries two different target miRNA sites (Figure 3C). One target location resides within the 3’ most UTR and is in a region of low conservation between human and tammar. The second location lies within a cryptic 3’UTR that is utilized in an alternatively-spliced isoform of this gene [33]. Similar to C17ORF49, this miRNA site is in a region of high conservation between tammar and human and accordingly may represent a conserved miRNA target site. This 3’UTR, unlike most 3’UTRs in tammar, is highly conserved with human across its entire length, confounding inferences regarding the conservation of specific miRNA target sites as the conservation of this portion of the transcript may be independent of any miRNA regulatory pathway. The miRNA identified for the cryptic 3’UTR target site was found limited to the pouch young brain miRNA pool, indicating this gene is under miRNA regulation specifically in that tissue. Interestingly, this gene codes for a splicing factor that is involved in alternative splicing of transcripts (reviewed in [34]). While it is interesting to speculate that the derivation of a miRNA regulated splicing pathway may have evolved in the tammar brain, leading to species-specific adaptation, a more exhaustive search within brain subregions in human and other mammalian species would be needed to confirm species-specificity.

### Genome defense and piRNAs

The annotation of the piRNAs in tammar was restricted to the testis due to technical difficulties with the ovary-specific library. However, we were able to confirm that while piRNAs in this species are predominantly derived from mobile elements, we found this pool was enriched for retrotransposons such as LINEs, SINEs, and LTR-elements. As in other species, there were several piRNA subgroups that were specific to *de novo* repeats identified in this species that are not conserved with opossum, platypus, mouse or human (Figure 4). Within this *de novo* pool was enrichment for tammar-specific LINEs and LTR-elements. Given the restriction of piRNAs to the germ line, and their role in genome defense and reproductive isolation [2,35], our discovery that a subset of piRNAs within the tammar are derived from novel repeats may provide an explanation to the long-standing mystery of Haldane’s Rule [35] within macropodid marsupials [36,37]. While macropodid marsupials can produce viable offspring, male F1 hybrids are sterile, following the tenets of Haldane’s Rule in which the heterogametic sex is adversely affected in interspecific crosses [35]. In addition, the genomes of macropodid marsupial F1 hybrids experience instability specifically associated with mobile elements [38-40]. Thus, we postulate that the rapid evolution of mobile DNA across macropodid marsupial species may result in an incompatibility within species hybrids that is manifest in the male germline as a result of expressed piRNA incompatibilities [2,14,41].

### crasiRNAs and centromeres

The final small RNA class that was annotated as part of the tammar genome project is the crasiRNAs. First discovered in the tammar [15], crasiRNAs were hypothesized to be derived from mobile elements resident within centromeres [18]. Our analyses represent the first full annotation of small RNAs in this class range and have identified several salient characteristics that demarcate this class from other small RNAs (reviewed in [42]). Across both tissues examined (testis and fibroblast cells), we find enrichment for mobile DNA progenitor sequences (Figure 5). Unlike the piRNAs, the predominant class of element within crasiRNAs is the SINE retroelement, including a recently discovered SINE class, SINE28, although the distribution of SINEs within each pool is different between testis and fibroblast cells. Our analyses of specific members within the crasiRNAs cytologically confirm that progenitor sequences are enriched at centromeres (Figure 6, Additional file 4: Figure S1). Moreover, these progenitor sequences are enriched in CENP-A containing nucleosomes, further supporting the classification of these small RNAs as centromere-repeat associated. While it cannot be ruled out that discontinuous palindromic signature identified in the crasiRNAs is a feature of the progenitor sequence from which the crasiRNAs are derived, it may also be a pattern involved in the biogenesis and/or targeting of crasiRNAs within centromeric sequences.

While this study has provided sequence annotation and genomic location for these small RNAs, their function within the genome has yet to be determined and remains largely inferential. The fact that crasiRNAs are found specifically in CENP-A rich regions of the centromere points to a role in centromere function; how these small RNAs participate in the demarcation of CENP-A nucleosomes or in centromere function is unknown. Histone tail modifications are dynamic processes that are modulated by other protein complexes and noncoding RNAs, such as small RNAs. For example, it has been proposed that RNAs mediate the pairing of centromere-specific DNAs to chromodomain-like adaptor proteins which in turn recruit histone methyltransferases (HMTases) that target the H3K9 residue for methylation. This interaction may be stabilized by the centromere-specific heterochromatin protein 1 (HP1)[43,44]. The methylation of H3K9 also triggers DNA methylation of CpG residues in centromeres [45,46].

The role of RNA in the process of histone modification is not clear; however, regions of the genome once thought of as “junk”, such as repeated DNAs and centromeres, are transcriptionally active and can modulate epigenetic states. Centromeres have long been thought to comprise noncoding and transcriptionally inactive DNA. Surprising new evidence suggests that eukaryotic centromeres produce a variety of transcripts. The transcription of satellites has been observed in numerous eukaryotic species across a broad range of phyla, from yeast to human. The wide-spread conservation of satellite transcription is consistent with a conserved regulatory role for these transcripts in gene regulation or chromatin modification [47].

These transcripts may function in one of four ways: 1) They may facilitate post-transcriptional gene regulation [48], potentially through the RNA-induced silencing complex (RISC). In this pathway, double stranded (ds) RNAs are cleaved into short interfering RNAs (siRNAs, 21 nucleotide double stranded RNAs) that, upon association with RISC, mediate native mRNA inactivation [49]. 2) They may participate in the RNA-induced transcriptional silencing complex (RITS), a pathway in which siRNAs are involved in heterochromatin recruitment [50,51]. 3) Alternatively, in a manner analogous to the Xist transcript in mammalian X-inactivation, they may recruit heterochromatin assembly factors such as HP1 [52], histone deacetylases, SET domain proteins and Polycomb group proteins [53]). 4) Lastly, they may regulate the movement of chromosomes through nuclear territories via association with specific chromocenters and “transcriptional factories” [54,55]. Although the mechanisms are unknown, evidence that satellite transcripts participate in heterochromatin assembly and/or nucleosome recruitment is accumulating.

## Conclusions

The international efforts of the tammar wallaby genome project have provided the opportunity to survey the major classes of small RNAs in this Australian marsupial model. Targeting multiple tissues in tammar pouch young, we have identified both conserved and novel miRNA producing genes in the tammar genome. We surveyed the genome for mature miRNA target genes, identifying both conserved targets as well as novel targets. Of these novel target genes, locations of mature miRNA binding sites represent both tammar-specific regions of low conservation across mammals, as well as regions of high conservation between human and tammar. Such comparisons point to the potential for the tammar as a model system to identify previously unknown miRNA regulated genes in other mammalian systems. While our analyses of the piRNAs was limited to the testis, tammar-specific repeats were identified that produce piRNAs, possibly as part of the gonad-specific genome defense network. Lastly, this study includes the first in depth analyses of the newest small RNA class, the crasiRNAs. Derived largely from repeat elements found at centromeres and associated with CENP-A nucleosomes, this pool of small RNAs is enriched for SINEs and exhibits a unique, discontinuous palindrome signature that may indicate a novel biogenesis mechanism. In summary, this study catalogs the major constituents of the small RNA repertoire of the tammar and, given the data herein, provides insight into the regulatory networks in which these small RNAs participate.

## Methods

### Animal tissues and cell lines

The tammar wallabies of Kangaroo Island origin, South Australia were held in the University of Melbourne breeding colony. All sampling techniques and collection of tissues conformed to Australian National Health and Medical Research Council (2004) guidelines and were approved by The University of Melbourne Animal Experimentation & Ethics Committees.

Tissues (brain, liver, testis, ovary, skin biopsies) were collected from day 124 post partum pouch young male (n=1) and female (n=1). All tissues were collected under RNase-free conditions and snap frozen in liquid nitrogen for storage at −80°C until use.

Tammar primary cells were prepared from a day 10 post partum pouch young skin biopsy. Briefly, the primary cells were cultivated in 50% DMEM (containing 10% fetal bovine serum) (Invitrogen, Melbourne, Australia) and 50% AmnioMax (Gibco, Carlsbad, USA,) containing 15% fetal calf serum.

### Library preparation and sequencing

Small RNA cloning was performed as described in [56]. Briefly, 40μg Trizol extracted total RNA from tammar brain, liver, testis, and pouch young fibroblast cells grown in culture was electrophoresed on a 15% denaturing polyacrylamide gel with γ-^32^P]-ATP end labeled 19-mer, 24-mer and 33-mer oligonucleotides. The bands corresponding to the miRNA fraction (19-24nt), piRNA (24-33nt) and crasiRNA fraction (35-45nt) were excised and ligated to an adenylated 3’ adapter (IDT, Inc.). The 3’ ligated RNA was electrophoresed on a 15% polyacrylamide gel and the bands corresponding to the ligated fractions (miRNA, piRNA, crasiRNA) were excised. A 5’ ligation reaction and subsequent polyacrylamide gel purification followed by reverse transcription and PCR was performed in preparation for Illumina sequencing. Sequencing was performed on an Illumina GAII according to the manufacturer’s protocol.

### Clipping and trimming

Before mapping each small RNA pool to the tammar genome, each small RNA pool was subject to sequence adaptor clipping and trimming. Adapter clipping was performed using a custom script which aligned the appropriate adapter to each read. If there was an alignment of 5 or more bases at the edge of the read, the aligned portion was removed, otherwise the whole read was removed. After adapter removal, for each pool any read which did not match the desired size for a specific pool of small RNA was removed. After filtering, a significant number of reads were removed due to a failure to pass the size selection criteria; this is likely due to low stringency during the library preparation size selection.

### Small RNA Analysis Pipeline

The miRNA pipeline (Additional file 6: Figure S3A) is designed to leverage high throughput small RNA sequencing technologies to confirm previously predicted miRNA genes and to improve the speed and accuracy of new miRNA gene identification and in silico validation. This is accomplished by using appropriate small RNA reads to narrow down the hairpin precursor search space. The presence of a computationally identified hairpin loop, and a sequenced small RNA gives greater confidence to the predicted genes than each signal would alone. An earlier version of this pipeline was published in two genome biology papers [23,24]. The general structure of the pipeline has remained relatively unchanged however the parameters used in the hairpin loop identification have evolved to provide more robust results. The pipeline is succinctly reiterated below focusing on the areas which have changed since previous publication.

#### Preprocessing

It is necessary to process the small RNA reads before they are utilized in the pipeline as described. In this study, the adapters were trimmed by searching for exact substrings of length 5 nt or more at the 3’ and 5’ end of the read. If a read did not have at least 5 bases from the 3’ end of the read, it was ignored. Next the reads were size selected for the expected RNA size in each pool.

#### Short read mapping

Mapping was performed using Bowtie [57], allowing for at most 1 mismatch. All valid alignments were reported, the bowtie parameters were: -v 1, and -a. While this introduces false positives, the hairpin loop prediction that follows (see below) further refines the dataset, thus compensating for this “loose” reporting parameter. All sequence data are held under accession number [NCBI GEO: GSE30372].

#### Hairpin loop identification

After mapping the mature miRNA against the genome, each position +− 50 bp is inspected for a hairpin loop structure. In order to do this we utilize the nRNAfold program which is part of the Vienna RNA package [58]. The following parameters were used with that tool: -p -d2 --noLP -P vienna1.8.4.par. After the structural alignment is computed the we ensure the presence of the unmatched loop, and that 75% of the bases in the stem are matched. We also ensure the sequenced miRNA aligns to the stem portion of the hairpin. The pipeline was designed such that after the short read mapping stage, all the analyses can be easily decomposed into independent components and run in parallel. This allows the user to run the tool on massive data sets without pre-filtering any alignments.

#### miRNA identification

If a read was found to be associated with a hairpin in the genome at least once, then it was annotated as hairpin-associated. The pipeline defines a sequenced small RNA as a bona fide miRNA gene only if it was annotated as hairpin-associated. All sequenced reads which were not bona fide were excluded from further analysis.

This pipeline is similar to mirDeep2 [59] and all predictions made by our pipeline were compared against the mirDeep2 pipeline for further confirmation. Our tool differs from mirDeep2 in two major ways. First mirDeep2 uses a pre-filtering step to filter out potential hairpins which do not have a predetermined number of sequence miRNA at each location. We chose to apply coverage filters after the pipeline was run because it is much more convenient in this type of exploratory data analysis. Secondly we do not provide a statistical score or a p-value for each of our predicted hairpins. Instead we indicate if the hairpin sequence was found in expressed mRNA.

#### Gene definition

An important part of identifying miRNA genes and miRNA targets is reliable gene annotation of the genome. Unfortunately the tammar genome is incomplete, as are the annotations. While several genes have been studied previously and have been annotated in depth, including introns, exons and flanking regions, the vast majority of gene annotations do not have such a well defined structure and therefore we employed the following convention to annotate the genome.

The Ensembl annotation was used to provide a foundation, however incomplete gene structures were expanded to approximate missing components. If a gene annotation was missing the 5’ and or 3’ flanking region, then the regional limits were expanded by 1000bp to approximate flanking UTRs. Of note, given that the majority of gene annotations do not contain internal structure, we were unable to delineate introns from exons in many cases.

All code used in the miRNA pipeline is available at https://bitbucket.org/jrl03001/mirid.

#### miRBase comparison

The miRBase database version 19 contains a collection of mature miRNA and hairpin precursor RNAs [25]. The hairpins of the putative miRNA genes were aligned against the hairpin collection of miRBase using nucmer with the following parameters: --maxmatch, --minmatch 15. The alignments were filtered to ensure that putative mature miRNA was found in the miRBase hairpin sequence with 95% identity. The best alignment was reported for each candidate. The miRBase ortholog identified is listed in Table 2 and Additional file 1: Table S1.

### piRNA and crasiRNA annotation

The pi and crasiRNA pools were annotated by first mapping the pools to the Meug_2.0 tammar genome assembly as described in the small RNA mapping section. Next, database predicted and *de novo* repeats were mapped to the genome using RepeatMasker. A small RNA was considered overlapping, or associated with a repeat, if at least one base pair overlapped with a repeat. The RNAs were allowed to map to multiple locations and therefore a single RNA could be annotated as derived from multiple repeats. This strategy allowed for some flexibility in small RNA annotations since repeat classes are often not distinct on a sequence level. SINE28 crasiRNA was validated via small RNA Northern analyses (Additional file 6: Figure S3B).

### Primed in situ hybridization

All primers (Additional file 7: Table S4) were designed from Repbase consensus sequences using default settings of Primer 3 and target regions represented in the crasiRNA pool. Metaphase chromosomes prepared from fibroblast cell lines were harvested and fixed to glass slides per standard methods. Briefly, colcemid was added to a final concentration of 0.1ug/mL at 37°C for 1–2 hours, cells were trypsinized and treated with 0.075M KCl at 37°C for 15–20 mins, pre-fixed, and fixed with 3:1 methanol:acetic acid (modified Carnoy’s). Cells were dropped onto acetone cleaned slides, air-dried overnight, dehydrated and stored at −20°C. A HybriWell™ reaction chamber (Schleicher & Schuell) was placed on the slide prior to denaturation at 93°C, at which point the reaction mixture was immediately applied. The reaction mixture consisted of 1μg each of primer, 1mM dCTP, dGTP, dATP, 0.01mM DIG-11-dUTP (Roche), 1X Taq-buffer (Promega), 4 units Taq polymerase (Promega), and distilled water to a final volume of 100μl. The reaction chamber was sealed, the slide placed on a Hybaid PCR Express In Situ Flat Block thermal cycler at 93°C for 3 mins followed by primer extension at 60°C for 10 minutes and extension at 72°C for 10 minutes. The reaction chamber was removed and the slide was placed in 55°C 0.2% SSC/0.2%BSA 2 x 5min. After blocking with 5% bovine serum albumin in 0.2% Tween 20/4XSSC (4XT), detection was performed using anti-digoxigenin fluorescein (sheep) (Roche) at 37°C in a humid chamber for 30 min. Excess detection reagents were washed at 45°C in 4XT. Slides were mounted in Vectashield + DAPI (Vector Labs).

### Small RNA Northern

The small RNA northerns were performed as per [15] with the following modifications: small RNAs less than 200bp were isolated using Ambion’s mirVana Isolation kit and 1 ug of size selected RNA was loaded onto the gel for each sample. After transfer, the membrane was chemically crosslinked as per [60]. An oligo corresponding to the most abundant miRNA read (miR20A: TAAAGTGCTTATAGTGCAGGTAG), let 7 as a control (ACTATACAACCTACTACCTCA), or a dsRNA derived from SINE28 (ACAAACCCTTGTGTCGAGGGCTGACTTTCAATAGATCGCAGCGAGGGA) was end labeled with P^32^ and hybridized at 58°C overnight. Stringent washes were performed at 2XSSC/0.1%SDS at room temperature and 2XSSC/0.1% SDS at 58°C.

### ChIP-seq library construction and sequencing

Tammar fibroblast cells were maintained at 35°C, 5%CO_2_ in Dulbecco’s modification of Eagle’s medium with penicillin-streptomycin (20units/20ug/mL), L-glutamine (1.46mg/mL), and supplemented with 10% fetal bovine serum (Atlanta Biologicals). Cells were harvested with trypsin-EDTA (Invitrogen) at 80% confluency and resuspended in phosphate buffered saline (PBS) to a concentration of 4 million cells/mL. Cells were crosslinked with formaldehyde at a final concentration of 1% for 10 minutes, rinsed twice with 500μl PBS and pelleted. Chromatin immunoprecipitation (ChIP) of pre-crosslinked cells was performed using the SOLiD ChIP-Seq Kit for the SOLiD 4 system per manufacturer’s protocol. Pelleted cells were lysed with lysis buffer containing protease inhibitors at a concentration of 1 million cells per 50μl for 10 minutes. Chromatin was sheared using the Covaris S2 with the following conditions: duty cycle: 5%, intensity: 2, cycles per burst: 200, cycle time: 60 seconds, cycles: 12, temperature: 4°C, power mode: frequency sweeping, degassing mode: continuous. Sheared chromatin size and quality was evaluated on a 2% agarose gel. Dynabeads (Invitrogen) and 10μg of custom tammar CENP-A antibody (Biosynthesis) were coupled overnight with rotation at 4°C. Sheared chromatin was diluted to 100,000 cells and 200,000 cells per 100μl dilution buffer with protease inhibitors and incubated with the coupled CENP-A antibody and Dynabeads at 4°C for two hours with end-over-end rotation. The immunoprecipitated chromatin was washed, reversed crosslinked, purified, and eluted as per the manufacturers protocol with the modification that DNA was incubated with the DNA Purification Magnetic Beads at room temperature for ten minutes instead of five. A no antibody control and an input DNA control were treated the same way. Sample quality was evaluated using the Quant-iT Picogreen Kit (Invitrogen). Real time PCR was used to assess the enrichment over background by using primers for KERV LTR. The primers were nULF (5^′^-TAKCTCGKGTATTTCMGCCTCTTC-3^′^) and nULR (5^′^-GGCTTTCCTGAYCCTACTTAARCYC-3^′^). Library construction and sequencing was performed with optimized libraries using the Applied Biosystems SOLiD 4 system and manufacturers protocols. All sequence data are held under accession number [NCBI GEO: GSE30372].

### ChIP-seq mapping and peak calling

Since CENP-A is a histone specific to the repeat-rich centromeres of the genome, a typical ChIP-seq mapping strategy was not employed. Under such a strategy, reporting only uniquely mapped reads would eliminate many of the repeat-associated reads (if not all), while reporting only one map location per read would underestimates the coverage. Conversely, reporting all mapped reads to the genome proved impossible due to disk space limitations. Instead, pericentromeric contigs were identified in Meug_2.0 using previously annotated centromere repeats [15,27]. ChIP-seq sequences were mapped against these contigs and each read was allowed to map to at most one location. While this strategy may over estimate the mapped depth, especially if the immunoprecipitation target sequences are present across all centromeres. Peaks were called using a model based approach MACS [61].

### crasiRNA motif

In order to quantify the observed palindromic motif and compare it to the miRNA pool, palindromic score and statistical significance functions were developed. The palindromic score function works as follows: for every instance of a small RNA aligning to the genome, the alignment plus 50 bases up and down stream were extracted. Small RNAs which aligned to the edge of a contig such that there were not 50 bases up and down stream were ignored. Each instance was tested for at least five distinct 3-mers to ensure it contained nontrivial information (i.e. not a simple repeat). The palindromic score of the window was calculated by computing the reverse complement of the window and looking at each position of complementary matches. The p-value of each score was computed empirically by randomizing the window 100 times and obtaining a palindromic score, thus ensuring that the base composition of the test was the same as the original. The p-value is the number of randomized windows which have a palindromic score equal to or greater than the original.

## Abbreviations

CENP: Centromere protein; KERV: Kangaroo endogenous retrovirus; Nt: Nucleotide; Kb: Kilobase; Bp: Base pair; UTR: Untranslated region; piRNA: Piwi interacting RNA; siRNA: Short interfering RNA; miRNA: micro RNA; rasiRNA: Repeat associated small interfering RNA; crasiRNA: Centromere repeat associated short interacting RNA; LINE: Long interspersed nuclear element; SINE: Short interspersed nuclear element; LTR: Long terminal repeat ; ChIP: Chromatin immunoprecipitation; ChIP-seq: Chromatin immunoprecipitation and deep sequencing; DAPI: 4',6-diamidino-2-phenylindole; PBS: Phosphate buffered saline; FBS: Fetal bovine serum; EDTA: Ethylenediaminetetraacetic acid.

## Competing interests

The authors declare that they have no competing interests.

## Authors’ contributions

JL and RO conducted the computational analyses. DC performed the small RNA sequencing on an Illumina instrument provided by GH. JB performed the PRINS experiments. LH and SQ performed the ChIP-seq. LH performed the centromere contig mapping. NJ and SM performed the small RNA Northerns. MR and AP performed tammar pouch young dissections. MO helped planning the selection analyses and writing. JL, DC, MO and RO conceived and wrote the study. All authors read and approved the final manuscript.

## Supplementary Material

Additional file 1: Table S1 Ensembl-predicted miRNA genes confirmed by our pipeline. Those with transcripts identified in tammar embryo transcriptomes are indicated, as are the miRNA genes confirmed by miRDeep2 and the miRBase orthologs. Click here for file

Additional file 2: Table S2 Complete annotations for all piRNAs in tammar testis. Annotation names based on RepBase entries. Click here for file

Additional file 3: Table S3 Complete annotations for all crasiRNAs in tammar fibroblast cells (A) and testis (B). Annotation names based on RepBase entries. Click here for file

Additional file 4: Figure S1 Primed in situ hybridization for localization of crasiRNA progenitor sequences, (green/red) to tammar metaphase chromosomes (grey). A. L1-2. B. L1-3. C. LTRX. D. LTR4. E. RTE2. Click here for file

Additional file 5: Figure S2 Screen capture from Broad institute Integrative Genomics Viewer (IGV) showing a tammar contig with mapping anti-CENP-A ChIP seq reads, crasiRNA reads and repeats as annotated by Repeat Modeler. Top of each panel are the coverage profiles and bottom (not shown in full detail) are alignment locations of individual reads. Click here for file

Additional file 6: Figure S3 A. Pipeline of the small RNA processing for miRNAs. The “small RNA reads” and “gene annotation” trapezoids represent the input to the miRNA pipeline. The “preprocess”, “map”, “hairpin identification” and “miRNA identification” blue boxes are the stages in the pipeline which filter out the true miRNA reads from the noise. Finally the miRNA genes and targets are identified from the hairpins, miRNA and gene annotations. Each of these steps is explained in detail in the methods section. B. Northern validation of (left) miRNA gene (miRNA20A) and (right) crasiRNA (SINE28). Click here for file

Additional file 7: Table S4 Primers used in PRINS. Click here for file
