# Supplementary material for: Unique small RNA signatures uncovered in the tammar wallaby genome
Source: BMC Genomics. 2012 Oct 17;13:559. doi: 10.1186/1471-2164-13-559 (PMC3576234; doi:10.1186/1471-2164-13-559)
Supplement: Additional file 1: Table S1 — Ensembl-predicted miRNA genes confirmed by our pipeline. Those with transcripts identified in tammar embryo transcriptomes are indicated, as are the miRNA genes confirmed by miRDeep2 and the miRBase orthologs. [file 1471-2164-13-559-S1.doc]

Supplemental Table 1

| miRNA count | Ensembl Meug 1.0 annotation | Symbol | Hairpin Alignment | mRNA | miR Deep | miBase |
| --- | --- | --- | --- | --- | --- | --- |
| 2081 | ENSMEUG00000017535 | miRNA gene | ..(((...))).....(((((......)))))..((((((..((((..(((((((((..(((((((.(((((............)))))))))))))))))))))..))))..)))))). | X | X | mdo-mir-181c |
| 1520 | ENSMEUG00000017079 | miRNA gene | .......((((((((.......))).))))).......((((.((.((.(((((((((...(((((((((((..............))))))))))).))))))))).)).)).)))).... | X |  | ggo-mir-30d |
| 1304 | ENSMEUG00000017109 | miRNA gene | .(((((((((((((((((..(((((((((((................)))))))))))))))))))))))))))).(((((........(((((.((.......)).))))).))))).... | X |  | eca-mir-30e |
| 1278 | ENSMEUG00000016917 | miRNA gene | ((((((((....................(((((((((((((((((((((((((.((...............))..)))))).....))))))))...)))))))))))))))))))... | X |  |  |
| 1276 | ENSMEUG00000017280 | miRNA gene | (((((((((((((((((.((((.(((((((...............)))))))))))))))))))))))))))).............((((....))))..((((...((.....)))))) | X |  | aca-mir-27b |
| 1276 | ENSMEUG00000017048 | miRNA gene | (((((((((((((((((.((((.(((((((...............)))))))))))))))))))))))))))).............((((....))))..((((...((.....)))))) | X |  | aca-mir-27b |
| 1011 | ENSMEUG00000017598 | miRNA gene | ....((((((.....(((....))).((((.((((((...)))))).))))..((((((..(((((((.((((.............))))))))))))))))))))).))........... | X |  | aca-mir-181a-1 |
| 1011 | ENSMEUG00000017378 | miRNA gene | ....((((((.....(((....))).((((.((((((...)))))).))))..((((((..(((((((.((((.............))))))))))))))))))))).))........... | X |  | aca-mir-181a-1 |
| 653 | ENSMEUG00000017566 | miRNA gene | (((((((((((((((((((((.((.(((((((...............))))))))).)))).)))))))))))))))))......(((((((......))).))))................. | X |  |  |
| 448 | ENSMEUG00000017661 | miRNA gene | ..(((.(((((((........)))..)))).))).(((((.((((((((.((((.(((((.((((((((((...............)))))))))).))))))))).)))))))).))))).. | X |  | aca-mir-10b |
| 195 | ENSMEUG00000017842 | miRNA gene | .......(((.(((..(((.((....)).))).))).)))((((((((..(((((((((((.((((((((((..................))))))))))))).))))))))..)))))))). | X |  | cgr-mir-34a |
| 56 | ENSMEUG00000017146 | miRNA gene | ..((((((((((......))).)))))))......(((...(((((((((..(((((((.(((((((((((............))))))))))).)))))))..)))))).)))..))). | X |  | bta-mir-122 |
| 46 | ENSMEUG00000016851 | miRNA gene | ......((.((((((((......)).)))))).))((((...(((((((.((((.(((((.((((((((((................)))))))))).))))))))).)))))))...)))). | X |  | age-mir-10a |
| 36 | ENSMEUG00000017831 | miRNA gene | ...........((.((((((..(((((((.((((.............))))))))))))))))).))(((((((((.(((((..(((.....)))....))))).))..)))))))..... | X |  | aca-mir-181a-2 |
| 36 | ENSMEUG00000016905 | miRNA gene | ..............(((.......))).........(((((((.....(((((((((..(((((((((((............)))))))))))))))))))).....)))))))...... | X |  | ccr-mir-181b |
| 28 | ENSMEUG00000017440 | miRNA gene | ....((.(((..((.((......)).)).))).))((..((((((((.((.((.((.(((.(((((((((.((............)))))))))))))))).)).)).)))))))).)). | X |  |  |
| 20 | ENSMEUG00000017742 | miRNA gene | .((((((((..((((.((.((((((((((((.............)))))))))))).)).)))).)))))))).(((((((........)).)))))..((((.(((......))).)))) | X |  | bta-mir-145 |
| 14 | ENSMEUG00000017240 | miRNA gene | (((((((((((((((.((((((((.(((.((..............)).))))))))))).))))))....))))))...)))....((.(((....))).))................... | X |  | sha-mir-199a |
| 12 | ENSMEUG00000017541 | miRNA gene | .....((((((((.........))))))))......((((((..(((((..(((((((((.((((.((((((.............)))))).)))).)))))))))..)))))..)))))) | X |  | mdo-mir-1546 |
| 12 | ENSMEUG00000017281 | miRNA gene | ..((.(((((((((((((((((((((((...((((............))))..)))))))))))))))))...........(((((.(((......)))..))))).....)))))).))... | X |  | bta-mir-101-2 |
| 10 | ENSMEUG00000017477 | miRNA gene | ((((...(((((((((.((((((((((((((((..............)))))))))))))))).)))))))))..))))((((((.(((((....)))))...(((...)))...)))))) | X |  | gga-mir-187 |
| 9 | ENSMEUG00000017545 | miRNA gene | ........((((((((........))))).)))..(((((((((((((.(((((((.(((.((((((((((............)))))))))).))).)))))))..))))))))))))).. | X |  | meu-mir-15c |
| 9 | ENSMEUG00000017368 | miRNA gene | ........((((((((........))))).)))..(((((((((((((.(((((((.(((.((((((((((............)))))))))).))).)))))))..))))))))))))).. | X |  | meu-mir-15c |
| 9 | ENSMEUG00000017286 | miRNA gene | ((.((((((.(((...(((((............))))).))).)))))).))((((((((((((((((((((((((((((.....))))))).........))))))))))))))))))))) | X |  | bta-let-7f-1 |
| 9 | ENSMEUG00000017259 | miRNA gene | ..(((.((((((((((((((.((.((((..((((.............)))))))).)).)))))))))))))).))).((.(((.((((.......(((....))).)))).)))))..... | X |  |  |
| 9 | ENSMEUG00000017229 | miRNA gene | ..((((((((((((((((((.((.((((..((((.............)))))))).)).))))))))))))))))))((((.......))))...... | X |  | bta-mir-148b |
| 8 | ENSMEUG00000017698 | miRNA gene | ..(((.(((((((((((.(((((((((....((((............))))))))))))).))))))).)))).))).(((((......((.((((((....)).)))))))))))...... | X |  | eca-mir-221 |
| 8 | ENSMEUG00000016964 | miRNA gene | .....((((((((((((((((((((((..................)))))..))))))))................)))))))))..((((((.......))))))............. | X |  |  |
| 8 | ENSMEUG00000016878 | miRNA gene | ..(((.(((((((((((.(((((((((....((((............))))))))))))).))))))).)))).))).(((((......((.((((((....)).)))))))))))...... | X |  | eca-mir-221 |
| 7 | ENSMEUG00000017744 | miRNA gene | ((.(((..(((...)))...)))))..........((((..((.(((((..(((((((((((((...(((((...............)))))..))))))))))))).))))).))..)))) | X |  |  |
| 7 | ENSMEUG00000017277 | miRNA gene | ..(((((.(((((((((((((((((((((.((((.............)))).))))))))))))).)))))))).))))).........((((..(((........)))..))))....... | X |  | mdo-mir-200a |
| 4 | ENSMEUG00000017828 | miRNA gene | .......(((..((.....))..)))((((((.....(((((.(((((..(((((((((.((.((((((((............)))))))).)).))))))))).)))))))))))))))). | X |  | mdo-mir-146a |
| 3 | ENSMEUG00000017730 | miRNA gene | .(((((.(((((..(((((((((((((((((.(((((..........))))))))))))))))))))))..))))).)))))...(((.((((((.............)))))).))).... |  |  |  |
| 3 | ENSMEUG00000016895 | miRNA gene | ...((((((((((((((..(((.(((..((((.......))))((((.((((....)))).((.(((........))).))...))))....))))))......)))))))))))))) |  |  |  |
| 3 | ENSMEUG00000017696 | miRNA gene | .......((((....))))..........(((((((((((((((.((..............)).))))))))...)))))))..(((...(((((((.....)))))))...))).... | X |  |  |
| 3 | ENSMEUG00000017668 | miRNA gene | .(((((.(((((..(((((((((((((((((.(((((..........))))))))))))))))))))))..))))).)))))...(((.((((((.............)))))).))).... |  |  |  |
| 3 | ENSMEUG00000017661 | miRNA gene | .((((((((.((((.(((((.((((((((((...............)))))))))).))))))))).))))))))....((((.........(((((....)))))........))))... | X |  | aca-mir-10b |
| 3 | ENSMEUG00000017516 | miRNA gene | .(((..((((((.....((.(((((.((((((....(((((((...................)))))))))))))))))).)).......(((......))).))).)))..))).... | X |  |  |
| 3 | ENSMEUG00000017498 | miRNA gene | ....(((.((.(((((((.((((((((((((((((((((...............))))).)))))))).....)))...)))).))))))).))............(((....)))))) |  |  |  |
| 3 | ENSMEUG00000017431 | miRNA gene | .....(((((((((..((.(((((((((..................)))))))))))..)))))))))....((.((((.((.........))...)))).))................. |  |  | eca-mir-363 |
| 3 | ENSMEUG00000017356 | miRNA gene | .....(((((((((..((.(((((((((..................)))))))))))..)))))))))....((.((((.((.........))...)))).))................. |  |  | eca-mir-363 |
| 3 | ENSMEUG00000017261 | miRNA gene | .....(((((((((..((.(((((((((..................)))))))))))..)))))))))....((.((((.((.........))...)))).))................. |  |  | eca-mir-363 |
| 3 | ENSMEUG00000016881 | miRNA gene | ..........((((((.(((((((((((((((...............))).))))))))).))).))))))..(((((.((((((.((..((.....)).)))))..)))))))).... | X |  | mdo-mir-338 |
| 2 | ENSMEUG00000017818 | miRNA gene | ......((((((((..(((((((((((..................)))))..))))))((....))..........))))))))...((((((.......))))))............. | X |  |  |
| 2 | ENSMEUG00000017769 | miRNA gene | ((((.((((((.(((((((((((((((.((((((.............)))))).))))))))))).)))).))))))))))(((((.....)))))...(((.((......))..)))..... |  |  | mdo-mir-200b |
| 2 | ENSMEUG00000017283 | miRNA gene | ....((((.((((((((.(((((((((((((..................))))))))))))).)))))))).))))(((((((((((........)))))))))))................ |  |  |  |
| 1 | ENSMEUG00000018321 | miRNA gene | .....((((((((((((((....((((((((....................)))))))).))))).))))((((((.((.((((((....)))))))).))))))((((...))))))))) | X |  |  |
| 1 | ENSMEUG00000018307 | miRNA gene | .......(((((((.(((((((((((.....................)).))).))))))))....)))))((((..((((((((((((.......))))...))))))))...)))). | X |  |  |
| 1 | ENSMEUG00000018298 | miRNA gene | .....((((((((((((((....((((((((....................)))))))).))))).))))((((((.((.((((((....)))))))).))))))((((...))))))))) | X |  |  |
| 1 | ENSMEUG00000018272 | miRNA gene | .......(((((((.(((((((((((.....................)).))).))))))))....)))))((((..((((((((((((.......))))...))))))))...)))). | X |  |  |
| 1 | ENSMEUG00000018253 | miRNA gene | .......(((((((.(((((((((((.....................)).))).))))))))....)))))((((..((((((((((((.......))))...))))))))...)))). | X |  |  |
| 1 | ENSMEUG00000018202 | miRNA gene | .......(((((((.(((((((((((.....................)).))).))))))))....)))))((((..((((((((((((.......))))...))))))))...)))). | X |  |  |
| 1 | ENSMEUG00000018141 | miRNA gene | .......(((((((.(((((((((((.....................)).))).))))))))....)))))((((..((((((((((((.......))))...))))))))...)))). | X |  |  |
| 1 | ENSMEUG00000018123 | miRNA gene | .......(((((((.(((((((((((.....................)).))).))))))))....)))))((((..((((((((((((.......))))...))))))))...)))). | X |  |  |
| 1 | ENSMEUG00000018106 | miRNA gene | .......(((((((.(((((((((((.....................)).))).))))))))....)))))((((..((((((((((((.......))))...))))))))...)))). | X |  |  |
| 1 | ENSMEUG00000018065 | miRNA gene | .......(((((((.(((((((((((.....................)).))).))))))))....)))))((((..((((((((((((.......))))...))))))))...)))). | X |  |  |
| 1 | ENSMEUG00000018028 | miRNA gene | .......(((((((.(((((((((((.....................)).))).))))))))....)))))((((..((((((((((((.......))))...))))))))...)))). | X |  |  |
| 1 | ENSMEUG00000018001 | miRNA gene | ......((((((((.(((....(((((...)))))...((((((((.((((..(((.((.(((((((............))).)).)).)))))..)))))))))))))))))))).))). | X |  |  |
| 1 | ENSMEUG00000017987 | miRNA gene | .......(((((((.(((((((((((.....................)).))).))))))))....)))))((((..((((((((((((.......))))...))))))))...)))). | X |  |  |
| 1 | ENSMEUG00000017975 | miRNA gene | .......(((((((.(((((((((((.....................)).))).))))))))....)))))((((..((((((((((((.......))))...))))))))...)))). | X |  |  |
| 1 | ENSMEUG00000017946 | miRNA gene | .......(((((((.(((((((((((.....................)).))).))))))))....)))))((((..((((((((((((.......))))...))))))))...)))). | X |  |  |
| 1 | ENSMEUG00000017905 | miRNA gene | .......(((((((.(((((((((((.....................)).))).))))))))....)))))((((..((((((((((((.......))))...))))))))...)))). | X |  |  |
| 1 | ENSMEUG00000017887 | miRNA gene | .......(((((((.(((((((((((.....................)).))).))))))))....)))))((((..((((((((((((.......))))...))))))))...)))). | X |  |  |
| 1 | ENSMEUG00000017883 | miRNA gene | .......(((((((.(((((((((((.....................)).))).))))))))....)))))((((..((((((((((((.......))))...))))))))...)))). | X |  |  |
| 1 | ENSMEUG00000017863 | miRNA gene | .((((..........))))((((((.......))))))..(((.((((((.(((((((((..((((((((....................))))))))..))))))))).)))))).))) | X |  | aca-mir-218-1 |
| 1 | ENSMEUG00000017758 | miRNA gene | ..((((...((((((((..(((((((((((((((.((.............)).)))))))))))))))..))))))))....))))....(((((((........))))))).......... |  |  |  |
| 1 | ENSMEUG00000017699 | miRNA gene | ...((((((((((((......)))..)))))))))...((((....))))((..(((((((((.(((.((((((.............)))))))))..)))......))))))..)).. | X |  |  |
| 1 | ENSMEUG00000017662 | miRNA gene | ...((.(((((.......))))))).......((((((....((((((((.(((((((((((.((((((((.............)))))))).))))))))))).))))))))))))))... | X |  |  |
| 1 | ENSMEUG00000017557 | miRNA gene | ......(((((.(((((.....))))).)))))(((((((.....))))).)).......(((..((((.(((((..............)))))..))))..))).............. | X |  |  |
| 1 | ENSMEUG00000017517 | miRNA gene | .......(((((...((......))...)))))...(((....((((.(((((((((((.((((((((((((.............))))))))))))))))))))))).))))...))) | X |  | aca-mir-135-2 |
| 1 | ENSMEUG00000017309 | miRNA gene | ..((((...((((((((..(((((((((((((((.((.............)).)))))))))))))))..))))))))....))))....(((((((........))))))).......... |  |  |  |
| 1 | ENSMEUG00000017236 | miRNA gene | .(((.((((........)))).)))............(((.((((((((((((((((((...((((((((((..............))))))))))..))))))))))))))))))))) |  |  | bta-mir-135b |
| 1 | ENSMEUG00000017138 | miRNA gene | (((((.(((..........((((((((((((((((((((((............))))))))))))))))))))))..........(((.((......)).)))))).)))))....... |  |  |  |
| 1 | ENSMEUG00000017087 | miRNA gene | ..((((...((((((((..(((((((((((((((.((.............)).)))))))))))))))..))))))))....))))....(((((((........))))))).......... |  |  |  |
| 1 | ENSMEUG00000016965 | miRNA gene | .((((((((((((..((((((.(((((.(((((...(((..............)))...))..))).)))))))))))..)))))....................))).))))...... | X |  |  |
| 1 | ENSMEUG00000016836 | miRNA gene | ..((((...((((((((..(((((((((((((((.((.............)).)))))))))))))))..))))))))....))))....(((((((........))))))).......... | X |  |  |
