# Supplementary material for: Unique small RNA signatures uncovered in the tammar wallaby genome
Source: BMC Genomics. 2012 Oct 17;13:559. doi: 10.1186/1471-2164-13-559 (PMC3576234; doi:10.1186/1471-2164-13-559)
Supplement: Additional file 2: Table S2 — Complete annotations for all piRNAs in tammar testis. Annotation names based on RepBase entries. [file 1471-2164-13-559-S2.doc]

Supplemental Table 2.

| **reads** | **element** | **reads** | **element** |
| --- | --- | --- | --- |
| 48947 | MAR1 | 31 | WALLSI3 |
| 32925 | WALLSI2 | 23 | Plat_L3 |
| 19722 | HAL1-3A_ME | 22 | P7SL_MD |
| 9637 | HAL1-3_ME | 18 | L1_Mars1b |
| 9094 | L1-2_ME | 14 | U5 |
| 4268 | RTE-2_ME | 12 | tRNA-Glu-GAG_ |
| 3228 | WALLSI1 | 11 | tRNA-Lys-AAG |
| 3093 | L1-4_ME | 11 | tRNA-Lys-AAA |
| 2720 | MIR3_MarsB | 11 | MarsTigger6 |
| 2389 | MIR3_MarsA | 9 | tRNA-Val-GTY |
| 1333 | LTRX_ME | 9 | L1_Mars1 |
| 1224 | L2_Mars | 7 | tRNA-Tyr-TAC |
| 925 | ERVII_ME_LTR | 6 | tRNA-Val-GTG |
| 771 | L1-1_ME | 5 | BovB_Mars |
| 679 | RTE-2_MD | 4 | tRNA-Gly-GGG |
| 647 | MIR_Mars | 4 | L1_Mars1a |
| 647 | DNAT_ME | 3 | U4 |
| 429 | WSINE1 | 3 | MamRep605 |
| 423 | ERVIIA_ME_LTR | 2 | tRNA-Ser-TCA(m) |
| 367 | WALLSI1A | 2 | Tigger1a_Mars |
| 358 | L2B_ME | 2 | MARINERNA1_ME |
| 298 | WALLSI4_Mar | 2 | HY1 |
| 211 | MdoRep1 | 2 | RETRO (denovo) |
| 203 | tRNA-Met-i | 1 | tRNA-Thr-ACA |
| 148 | RTE1_Mars | 1 | MER63_Marsup |
| 132 | LTR1N_MD | 1 | MamSINE1 |
| 118 | RTESINE2 | 1 | LTR200_MD |
| 112 | L1-4A_ME | 1 | L2-2_ME |
| 110 | WALLSI4 | 1 | Joey1 |
| 60 | LTR (*de novo*) | 1 | HY4 |
| 59 | LTR4_ME | 1 | Charlie4a_Marsup |
| 58 | SSU-rRNA_Hsa | 1 | Charlie4 |
| 42 | tRNA-Glu-GAA | 1 | Charlie1b_Mars |
| 32 | tRNA-Gly-GGY | 1 | DNA element (*de novo*) |
