# Supplementary material for: Unique small RNA signatures uncovered in the tammar wallaby genome
Source: BMC Genomics. 2012 Oct 17;13:559. doi: 10.1186/1471-2164-13-559 (PMC3576234; doi:10.1186/1471-2164-13-559)
Supplement: Additional file 3: Table S3 — Complete annotations for all crasiRNAs in tammar fibroblast cells (A) and testis (B). Annotation names based on RepBase entries. [file 1471-2164-13-559-S3.doc]

Supplemental Table 3A.

| **reads** | **element** | **reads** | **element** |
| --- | --- | --- | --- |
| 5199 | WALLSI2 | 12 | L2B_ME |
| 3981 | LSU-rRNA_Hsa | 11 | WALLSI1A |
| 3382 | SSU-rRNA_Hsa | 11 | tRNA-Thr-ACA |
| 1807 | MAR1 | 10 | tRNA-Leu-TTG |
| 1461 | HAL1-3A_ME | 10 | A-rich |
| 1292 | HAL1-3_ME | 9 | tRNA-Thr-ACY |
| 719 | tRNA-Gly-GGA | 9 | RTE-2_MD |
| 603 | tRNA-Lys-AAA | 8 | tRNA-Asn-AAC |
| 472 | L1-2_ME | 8 | HY3 |
| 344 | tRNA-Met-i | 7 | tRNA-Ile-ATA |
| 210 | SINE28 | 7 | LTR90B |
| 206 | RTE-2_ME | 7 | HY5 |
| 176 | tRNA-Glu-GAG_ | 6 | tRNA-Gln-CAA_ |
| 164 | tRNA-Val-GTY | 6 | MIR3_MarsA |
| 155 | tRNA-Val-GTG | 6 | L1_Mars1b |
| 130 | L1-1_ME | 6 | 5S |
| 93 | L1-4_ME | 5 | tRNA-SeC(e)-TGA |
| 84 | WSINE1 | 5 | tRNA-Cys-TGY |
| 51 | tRNA-Gly-GGY | 5 | HY4 |
| 50 | (TCTA)n | 4 | WALLSI4_Mar |
| 47 | (TAGA)n | 4 | U14 |
| 44 | tRNA-Lys-AAG | 4 | tRNA-Pro-CCA |
| 42 | P7SL_MD | 4 | MamRep605 |
| 34 | HY1 | 3 | tRNA-Leu-CTY |
| 24 | tRNA-Glu-GAA | 3 | RTE1_Mars |
| 23 | tRNA-Tyr-TAC | 3 | MIR_Mars |
| 23 | L1-4A_ME | 3 | L1_Mars1a |
| 20 | tRNA-Val-GTA | 2 | U5 |
| 20 | tRNA-Asp-GAY | 2 | LTR4_ME |
| 20 | MdoRep1 | 2 | LTR1N_MD |
| 18 | tRNA-Ala-GCG | 1 | WALLSI4 |
| 15 | tRNA-Gly-GGG | 1 | U4 |
| 15 | MIR3_MarsB | 1 | tRNA-Leu-CTG |
| 14 | U1 | 1 | Plat_L3 |
| 14 | tRNA-His-CAY_ | 1 | L2-2_ME |
| 13 | L2_Mars | 1 | Charlie4a_Marsup |

Supplemental Table 3B.

| **reads** | **element** | **reads** | **element** | **reads** | **element** |
| --- | --- | --- | --- | --- | --- |
| 138251 | WALLSI2 | 152 | L2B_ME | 7 | tRNA-His-CAY_ |
| 70701 | HAL1-3A_ME | 150 | MIR3_MarsA | 7 | Joey1 |
| 26313 | HAL1-3_ME | 105 | L1-4A_ME | 6 | U13 |
| 19425 | RTE-2_ME | 100 | tRNA-Ile-ATA | 6 | tRNA-Asn-AAC |
| 16120 | L1-2_ME | 92 | RTE1_Mars | 6 | A-rich |
| 11308 | LSU-rRNA_Hsa | 81 | MIR_Mars | 5 | tRNA-Thr-ACA |
| 8955 | MAR1 | 76 | tRNA-SeC(e)-TGA | 4 | WALLSI3 |
| 8011 | SSU-rRNA_Hsa | 65 | L1_Mars1b | 4 | tRNA-Pro-CCG |
| 6506 | tRNA-Lys-AAA | 59 | WALLSI4_Mar | 4 | MER63_Marsup |
| 5162 | L1-1_ME | 53 | LTR1N_MD | 3 | tRNA-Met_ |
| 4367 | WALLSI1 | 48 | tRNA-Val-GTA | 3 | LTR4_ME |
| 3301 | ERVIIA_ME_LTR | 44 | MdoRep1 | 3 | DNAT_ME |
| 3168 | ERVII_ME_LTR | 38 | HY4 | 2 | tRNA-Thr-ACY |
| 3097 | tRNA-Val-GTY | 37 | tRNA-Gly-GGG | 2 | tRNA-Ile-ATT |
| 2745 | tRNA-Val-GTG | 36 | U1 | 2 | tRNA-Gln-CAA_ |
| 2503 | tRNA-Gly-GGA | 31 | tRNA-Leu-TTG | 2 | tRNA-Arg-CGY_ |
| 1777 | tRNA-Glu-GAG_ | 28 | tRNA-Cys-TGY | 2 | tRNA-Arg-AGG |
| 1398 | tRNA-Met-i | 28 | RTESINE2 | 2 | Tigger1a_Mars |
| 757 | tRNA-Glu-GAA | 25 | WALLSI4 | 2 | Tigger15a |
| 684 | L1-4_ME | 23 | U2 | 2 | CheshMITE |
| 635 | RTE-2_MD | 21 | L1_Mars1 | 1 | UCON28a |
| 586 | SINE28 | 21 | HY5 | 1 | U4 |
| 538 | LTRX_ME | 21 | Charlie4a_Marsup | 1 | U14 |
| 482 | HY1 | 20 | L2-2_ME | 1 | tRNA-Gln-CAG |
| 460 | WSINE1 | 19 | tRNA-Ala-GCG | 1 | tRNA-Arg-CGA_ |
| 436 | tRNA-Leu-CTG | 12 | Plat_L3 | 1 | Tigger16a |
| 430 | (GGAGA)n | 12 | L1_Mars1a | 1 | Tigger1 |
| 393 | tRNA-Gly-GGY | 11 | tRNA-Tyr-TAC | 1 | MarsTigger5a |
| 379 | tRNA-Lys-AAG | 11 | 5S | 1 | MARINERNA1_ME |
| 374 | WALLSI1A | 10 | tRNA-Pro-CCY | 1 | LTR14_ME |
| 314 | (TCTCC)n | 10 | tRNA-Pro-CCA | 1 | L1MEf |
| 301 | MIR3_MarsB | 10 | MarsTigger6 | 1 | Charlie23a |
| 273 | tRNA-Asp-GAY | 10 | LTR200_MD | 1 | Charlie1a_Marsup |
| 267 | L2_Mars | 10 | Charlie4b_Marsup | 1 | Charlie1 |
| 234 | P7SL_MD | 9 | BovB_Mars |  |  |
| 162 | MamRep605 | 8 | HY3 |  |  |
