# Supplementary figures and images for: Unique small RNA signatures uncovered in the tammar wallaby genome
Source: BMC Genomics. 2012 Oct 17;13:559. doi: 10.1186/1471-2164-13-559 (PMC3576234; doi:10.1186/1471-2164-13-559)

Supplemental Figure 1

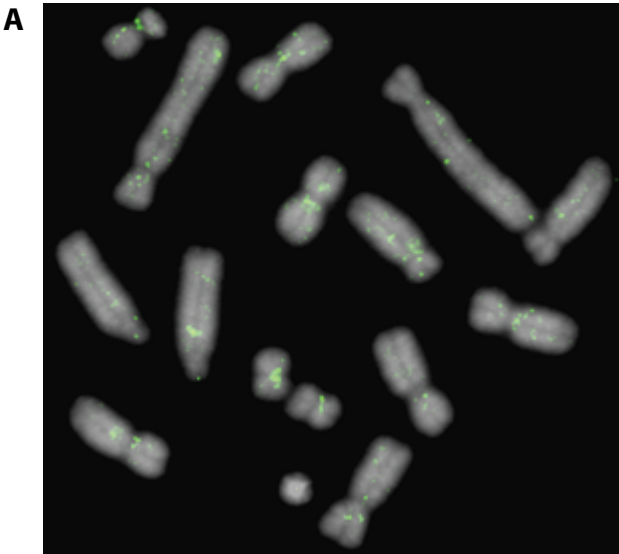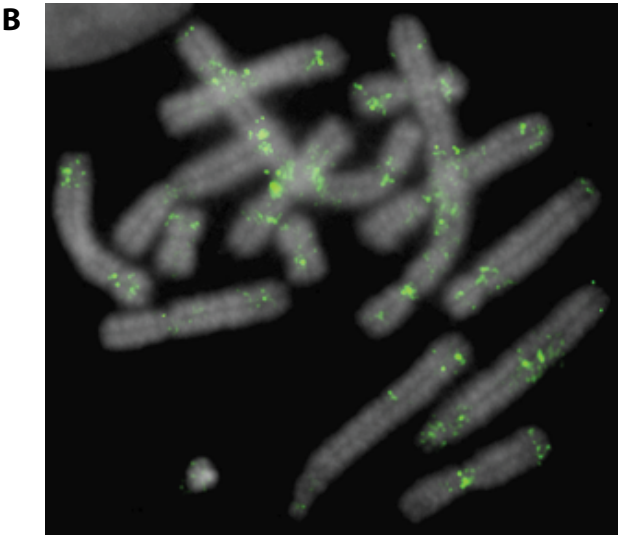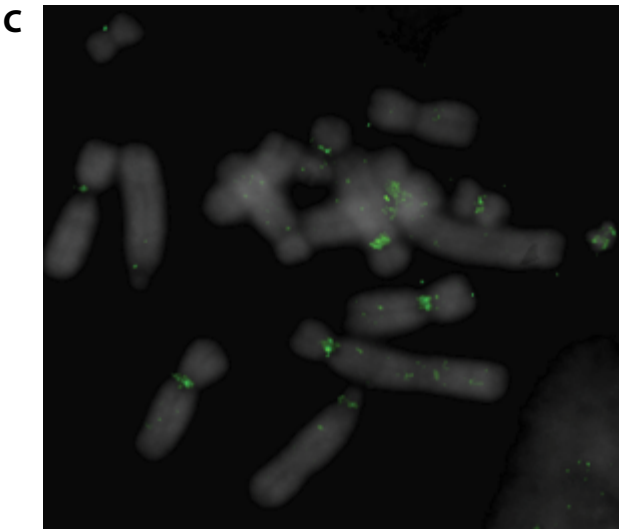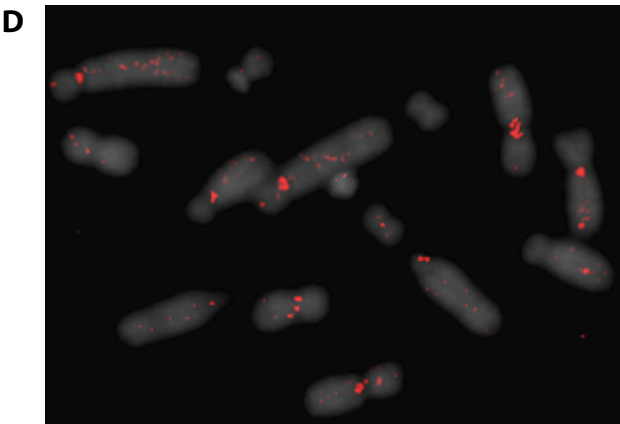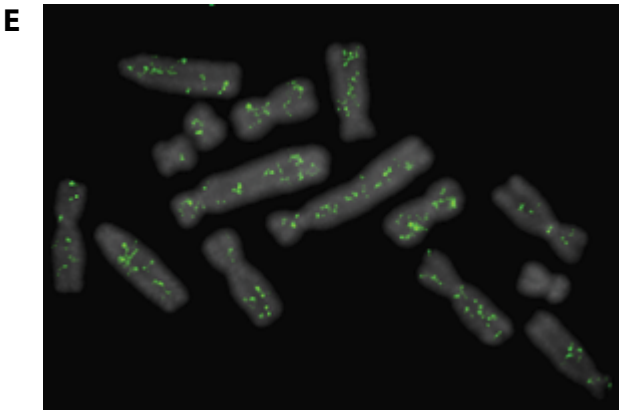

Supplement: Additional file 4: Figure S1 — Primed in situ hybridization for localization of crasiRNA progenitor sequences, (green/red) to tammar metaphase chromosomes (grey). A. L1-2. B. L1-3. C. LTRX. D. LTR4. E. RTE2. [file 1471-2164-13-559-S4.pdf]

Supplemental Figure 2

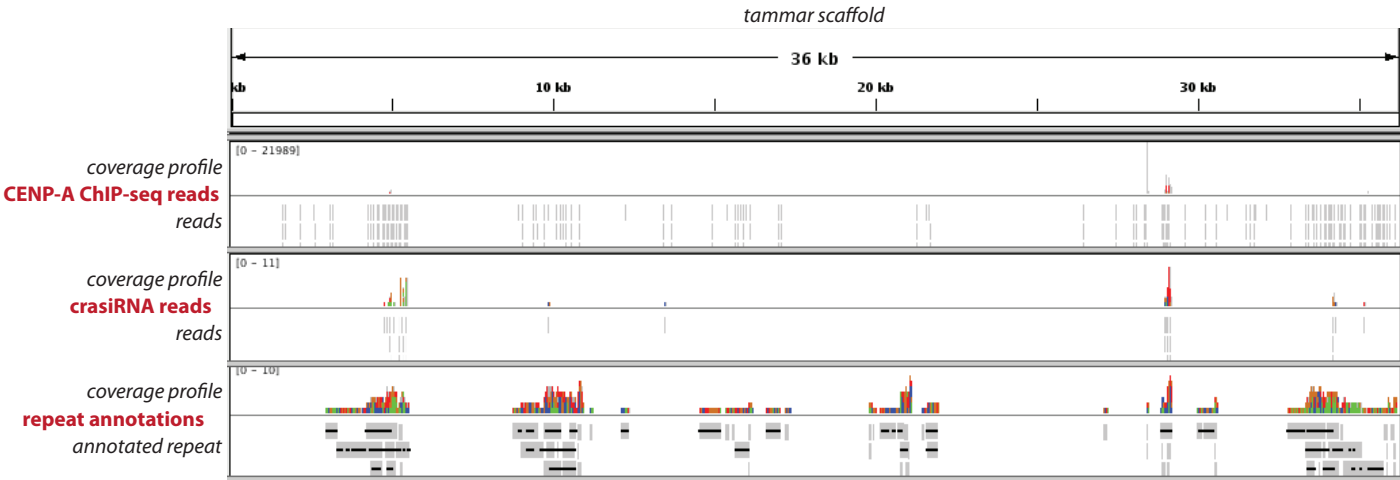

Supplement: Additional file 5: Figure S2 — Screen capture from Broad institute Integrative Genomics Viewer (IGV) showing a tammar contig with mapping anti-CENP-A ChIP seq reads, crasiRNA reads and repeats as annotated by Repeat Modeler. Top of each panel are the coverage profiles and bottom (not shown in full detail) are alignment locations of individual reads. [file 1471-2164-13-559-S5.pdf]

**Supplemental Figure 3A**

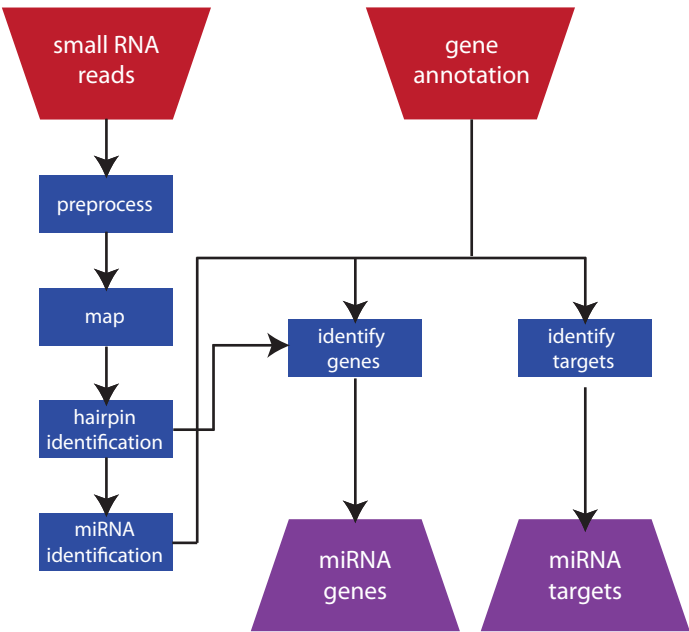

**Supplemental Figure 3B**

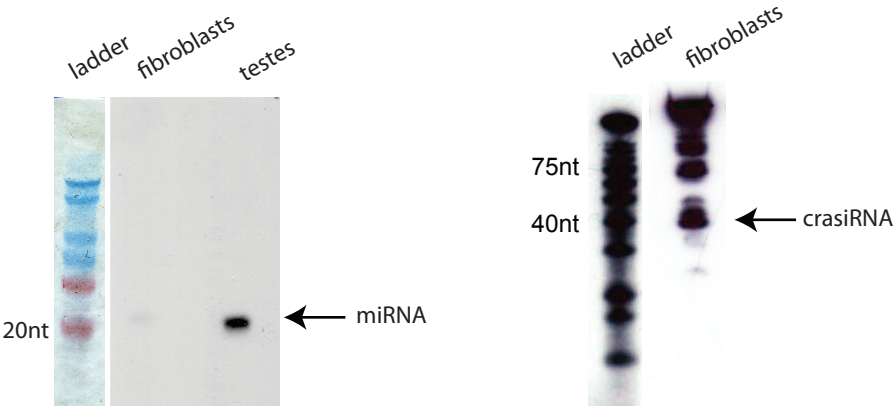

Supplement: Additional file 6: Figure S3 — A. Pipeline of the small RNA processing for miRNAs. The “small RNA reads” and “gene annotation” trapezoids represent the input to the miRNA pipeline. The “preprocess”, “map”, “hairpin identification” and “miRNA identification” blue boxes are the stages in the pipeline which filter out the true miRNA reads from the noise. Finally the miRNA genes and targets are identified from the hairpins, miRNA and gene annotations. Each of these steps is explained in detail in the methods section. B. Northern validation of (left) miRNA gene (miRNA20A) and (right) crasiRNA (SINE28). [file 1471-2164-13-559-S6.pdf]
