# Supplementary material for: Unique small RNA signatures uncovered in the tammar wallaby genome
Source: BMC Genomics. 2012 Oct 17;13:559. doi: 10.1186/1471-2164-13-559 (PMC3576234; doi:10.1186/1471-2164-13-559)
Supplement: Additional file 7: Table S4 — Primers used in PRINS. [file 1471-2164-13-559-S7.doc]

**Supplemental Table 4.**

| Primer Name | Forward Sequence | Reverse Sequence |
| --- | --- | --- |
| LTR4 | gaacccccttgaactctcct | gttgaggtttaggggtgctg |
| LTRX | caagcaccccaacatacaca | aaagatcttccccacccatt |
| SINE28 | ccttgtgtcgagggctgactt | gttcgtgtggaacctggcgctaaac |
| 5L1-2 | gcacaagaattgtgggacag | tggttgtagtcccagctcct |
| L1-3 | agtcactggctgggaaaatg | gctgggtaggtgattcttgg |
| RTE-2 | cactcaatgcagcctctgaa | ggaccaaaatgacatcacca |
